# The Effects of SNPs in the *LCORL* and *LTBP2* Genes on Comprehensive Performance Traits in Charolais Sheep

**DOI:** 10.1155/vmi/7393976

**Published:** 2026-08-27

**Authors:** Jiaqi Ge, Ziwen Qi, Jianing Xin, Yvxin Shi, Wangshu Li, Dakun LYu, Yunlong Guo, Fengzhi Liu, Decui Xia, Xuehai Du, Guochun Wang, Shuhuan Yang, Zeying Wang

**Affiliations:** ^1^ College of Animal Science and Veterinary Medicine, Shenyang Agricultural University, Shenyang, 110866, China, syau.edu.cn; ^2^ Life Engineering College of Shenyang Institute of Technology, No. 1 East Section of Binhe Road Shenfu Demonstration Zone 113122, Shenyang, Liaoning, China; ^3^ Fuxin City Animal Disease Prevention and Control Center, Fuxin, 123006, China; ^4^ Liaoning Agricultural and Rural Development Service Center, No. 7-1 Lingyuan Street Huanggu, Shenyang 110032, Liaoning, China

**Keywords:** growth performance, *LCORL*, *LTBP2*, meat quality performance, slaughter performance

## Abstract

The Charolais sheep is a world‐renowned breed of meat sheep, known for its large frame, heavy muscling, and rapid growth. A novel breed, the Chao Mu meat sheep, has been developed by utilizing the Charolais sheep as the paternal line. This advancement has significantly contributed to the genetic enhancement and breeding of indigenous meat sheep in China. However, body size varies among different Charolais families introduced in China. To address this, we employed DNA‐seq combined with MLR analysis to identify key genes associated with growth, slaughter, and meat quality performance. Isolation of genomic DNA was performed from blood samples obtained from 1400 Charolais sheep with recorded production performance data for subsequent statistical analyses of genotypes and haplotypes. A SNP (T136210A) was identified in *LCORL*, and another (G105452A) in *LTBP2*, in Charolais sheep. In the haplotype combinations of these genes, for growth, the dominant haplotype in ewes was TTAA. Multiple linear regression demonstrated a significant linear relationship between body weight and these performance metrics. BW = 0.690CC + 0.522LL + 1.546CW + 1.863WH − 2.386BaH + 1.613BH − 0.610BL + 3.422CaC − 0.819SH + 1.555FW − 0.575CD + 0.227HH − 118.193 (*R* ^2^ = 0.794) in ewes and BW = 1.472CC + 0.509HW − 88.303 (*R* ^2^ = 0.961) in rams. The TT (*LCORL*) in ewes and GG (*LTBP2*) in ewe lamb genotypes are promising candidates as molecular markers for the evaluation of growth performance. Genotypes TT (*LCORL*) and GG (*LTBP2*) were associated with slaughter performance, suggesting their potential utility as molecular markers.

## 1. Introduction

Originating in France, the Charolais sheep is a globally recognized large meat breed developed by crossing British Leicester and Southdown rams with local fine‐wool ewes [1]. The Charolais sheep breed is characterized by its uniformly white fleece, pink or gray facial pigmentation, large body frame, pronounced muscling, rapid growth rate, and stable genetic profile [2]. Nevertheless, body size traits exhibit a considerable variation across different populations of Charolais sheep, which may be attributed to divergence among family lines. In recent years, research on germplasm resources has intensified, driven by the goals of fixing superior genes and developing enhanced varieties. Concurrently, rising living standards have led to increasing market demand for lamb [3]. However, meat sheep are characterized by a long production cycle and low reproductive efficiency [4]. To address these constraints and meet market demands, one promising strategy is to select for larger body size through the identification and fixation of genetic variants associated with growth traits at the individual level.

Ligand‐dependent nuclear receptor corepressor‐like (*LCORL*, also referred to as *Mlr1*) is encoded by a gene located within the cell nucleus. This protein may function as a transcription activator that binds to the specific DNA element 5′‐CCCTATCGATCGATCTCTACCT‐3′ and has been closely associated with spermatogenesis [5, 6]. *LCORL* is predominantly expressed in the testis, specifically within meiotic spermatocytes, where it plays a central role in spermatogenesis. It functions by initiating and regulating the expression of specific genes in spermatocytes, thereby promoting their entry into meiosis. Both *LCORL* and *LCORs* (also referred to as *Mlr2*) are coexpressed in several tissues, suggesting potential synergistic interactions [6]. The identification by Al‐Mamun et al. [7] of *LCORL* on chromosome 6, which resides in a genomic region highly conserved across mammals and associated with body size variation, positions it as a potential regulator of body weight (BW) in sheep. Another study identified 90 DNA variants located upstream of *LCORL*, which are associated with asthma and exhibit sex‐specific effects, particularly contributing to disease susceptibility in males [8]. In Rambouillet sheep, F_ST_‐based analysis led to the identification of a large, highly conserved ROH island, within which *LCORL* was associated with key economic traits including milk production, growth, and carcass yield [9].

Latent transforming growth factor β‐binding protein 2 (*LTBP2*) belongs to the *LTBP* family. As the largest member of this family, *LTBP2* is an extracellular matrix protein characterized by a multidomain structure that facilitates its interaction with fibrillin microfibrils [10]. Given its high expression in elastic tissues such as the lung and aorta, emerging evidence suggests that serum *LTBP2* levels in patients presenting with acute dyspnea may serve as a novel biomarker for predicting all‐cause mortality [11, 12]. Shipley and colleagues investigated the age‐dependent expression patterns of *LTBP2* in rodents and demonstrated that *LTBP2* serves a structural role during embryo implantation. Their findings indicate that inactivation of this gene results in embryonic lethality during early development [13]. Meanwhile, *LTBP2* is secreted into the bloodstream during fibroblast differentiation. Comparative analysis of tissue staining and serum samples showed that both the rate of *LTBP2*‐positive staining in lung tissue and the concentration of *LTBP2* in serum were significantly elevated in patients with idiopathic pulmonary fibrosis compared to healthy controls [14]. Furthermore, *LTBP2* is proposed as a positional candidate gene that increases thoracic vertebral and rib number in pigs by suppressing Gdf11 activation [15, 16]. In a comprehensive overview of sheep meat production traits, *LTBP2* emerges as a key candidate gene with a fundamental influence on vertebral and rib number, as demonstrated in breeds including large‐fat‐tailed and Altay sheep [17].

The experimental protocol consisted of primer design, amplification of the target products, single‐nucleotide polymorphism (SNP) detection, and data analysis. All statistical analyses were carried out with SHEsis, SPSS, and conventional statistical methods. This study aimed to investigate polymorphisms in the *LCORL* and *LTBP2* genes and assess their associations with growth and slaughter traits in Charolais sheep. By analyzing genetic diversity and assessing trait correlations, this study seeks to elucidate the genetic determinants of performance, specifically identifying key genotypes and haplotype combinations. The findings will facilitate optimized gene selection and mating strategies for Charolais sheep, thereby enhancing core production performance. This study provides data‐driven models and decision‐making support for the industry to develop high‐yield, high‐quality, and efficient Charolais sheep populations.

## 2. Materials and Methods

### 2.1. Experimental Animals

A total of 1400 healthy and uniformly fed Charolais sheep were selected from the core breeding farm of Charolais sheep in Liaoning Province. All experimental procedures involving animals were approved by the Laboratory Animal Management Committee of Shenyang Agricultural University and conducted in compliance with its guidelines (Approval No. 2023041610). Venous blood (1 mL) was collected from the jugular vein of each sheep by a licensed veterinarian for subsequent DNA extraction. Following collection, transfer the blood into EDTA‐containing tubes and store at −20°C.

### 2.2. Production Data

Body size data were acquired using an intelligent phenotyping system for sheep. Slaughter data were collected following standard livestock and poultry slaughter operating procedures (GB/T43562‐2023). Meat quality data were determined according to the technical specification for the determination of sample meat quality (T/CAAA 102‐2023).

### 2.3. Definitions of Growth, Slaughter, and Meat Quality Traits

1. BW: live weight of experimental sheep after 24 h of fasting at the corresponding growth stage.
2. Body height (BH): vertical distance from the highest point of the withers to the ground when the sheep stands naturally.
3. Back height (BaH): vertical distance from the highest point of the 6th‐7th thoracic spinous process to the ground.
4. Waist height (WH): vertical distance from the highest point of the 3rd‐4th lumbar spinous process to the ground.
5. Sacral height (SH): vertical distance from the highest point of the sacrum to the ground.
6. Hip height (HH): vertical distance from the highest point of the ischial tuberosity to the ground.
7. Forehead width (FW): maximum horizontal distance between the upper edges of the two orbits.
8. Cannon circumference (CaC): circumference at the narrowest part of the left fore cannon bone.
9. Chest circumference (CC): circumference around the chest along the posterior edge of the scapula.
10. Limb length (LiL): vertical distance from the upper edge of the carpal bone to the ground.
11. Leg length (LL): vertical distance from the upper edge of the tarsal joint to the ground.
12. Body length (BL): straight‐line distance from the anterior edge of the shoulder point to the posterior edge of the rump.
13. Chest depth (CD): vertical distance from the highest point of the withers to the lower edge of the sternum.
14. Chest width (CW): maximum horizontal distance between the two shoulder joints.
15. Waist angle width (WAW): maximum horizontal distance between the outer edges of the two coxal tuberosities.
16. Hip width (HW): maximum horizontal distance between the outer edges of the two ischial tuberosities.
17. Preslaughter live weight (PLW): live weight after 24 h of fasting and 2 h of water deprivation before slaughter.
18. Carcass weight (CaW): weight of the body after bleeding, removing head, hooves, skin, and viscera, with kidneys and perirenal fat retained.
19. Bone weight (BoW): total weight of all bones after boning the carcass.
20. Dressing percentage (DP): percentage of CaW relative to live weight before slaughter.
21. Net meat percentage (NMP): percentage of net meat weight (after removing bones and fat) relative to live weight before slaughter.
22. Eye muscle area (EMA): muscle area measured on the cross section of the longissimus dorsi between the 12th and 13th ribs.
23. GR value: total thickness of soft tissue measured at the 12th‐13th rib, 11 cm from the dorsal midline.
24. Backfat thickness (BT): thickness of subcutaneous fat above the longissimus dorsi at the 12th‐13th rib.
25. Dry matter: percentage of dry matter weight relative to fresh sample weight after drying at 105°C to constant weight.
26. Protein content: crude protein content in muscle, expressed as a percentage of fresh sample weight, determined by the Kjeldahl method.
27. Fat content: crude fat content in muscle, expressed as the percentage of fresh sample weight, determined by Soxhlet extraction.
28. Ash content: percentage of ash weight relative to fresh sample weight after incineration at 550°C to constant weight.
29. Drip loss: percentage of weight loss after suspending muscle samples at 4°C for 24 h.
30. Cooked meat rate: percentage of cooked meat weight relative to fresh meat weight after heating to a core temperature of 70°C and cooling.
31. Meat color: *L* ^∗^ (lightness), *a* ^∗^ (redness), and *b* ^∗^ (yellowness) values measured on the cross section of the longissimus dorsi using a colorimeter.
32. Shear force: shear force value of cooked meat samples along the muscle fiber direction measured by a texture analyzer.
33. pH: pH value of the longissimus dorsi measured at 45 min and 24 h postmortem.

### 2.4. DNA Isolation

Add the blood (200 μL) to a 1.5‐mL centrifuge tube, followed by the addition of proteinase K (20 μL), and mix thoroughly. Then, add Buffer DL (200 μL), vortex to mix, and incubate the sample in a water bath at 56°C for 10 min. After incubation, add absolute ethanol (200 μL) to the mixture and invert the tube several times to ensure thorough mixing. The lysate was transferred to an adsorption column, incubated at room temperature for two minutes, and then centrifuged at 10,000 rpm for 1 min. Discard the flow‐through from the collection tube. Reinsert the adsorption column into the collection tube, add GW Solution (500 μL) to the column, and centrifuge at 10,000 rpm for 30 s. Discard the flow‐through. The column was placed back into the collection tube, to which wash solution (700 μL) was added, followed by centrifugation at 10,000 rpm for 30 s and discarding the waste. Repeat this washing step once more. After the final wash, discard the flow‐through and centrifuge the empty column at 12,000 rpm for 2 min. Following air‐drying at room temperature with the cap open, the DNA was eluted by applying CE buffer (50 μL) to the column, incubating for 3 min, and centrifuging at 12,000 rpm for 2 min at room temperature into a fresh 1.5‐mL centrifuge tube. Collect the resulting DNA solution. Following DNA extraction, the quality of all samples was assessed using a NanoDrop spectrophotometer. Only samples with an A260/A280 ratio between 1.8 and 2.0 and a concentration of at least 50 ng/μL were selected for subsequent experiments and stored at −20°C until further analysis.

### 2.5. Primer Design

The nucleotide sequences of the *LCORL* and *LTBP2* genes were retrieved from the NCBI database (RefSeq: NC_056059.1, NC_056060.1). Gene‐specific primers were designed using Primer Premier 5 software (Table 1).

**TABLE 1:** Primers used for amplification and genotyping of target regions in the *LCORL* and *LTBP2* genes.

Gene	Sense primer	TM (°C)	Antisense primer	TM (°C)	Fragment size (bp)	Regions
*LCORL*	5′‐CATACATTATTCCCACTTTC‐3′	47.2	5′‐GAAGCAGGACGAGTAGAG‐3′	46.6	840	135901–136740 (NC_056059.1)
*LTBP2*	5′‐AACACCGCCAGGAACTCA‐3′	56.4	5′‐TCCCACTTCGGGTCACAG‐3′	56.6	692	105241–106080 (NC_056060.1)

### 2.6. PCR Amplification

**FIGURE 1:**
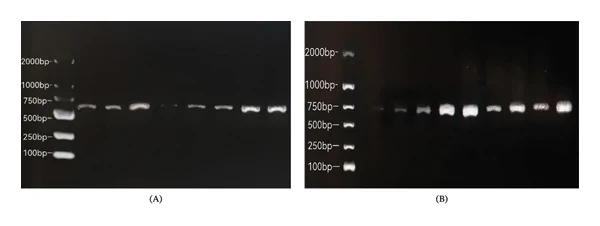
Electrophoretic analysis of PCR‐amplified fragments for *LCORL* (A) and *LTBP2* (B).

A 50‐μL PCR reaction system was prepared, consisting of 2× SanTaq PCR Mix (25 μL), forward primer (2 μL), reverse primer (2 μL), DNA template (1 μL), and ultrapure water (20 μL). The reagents were combined in a PCR tube in the order listed, thoroughly mixed, and centrifuged. Amplification was carried out under the following conditions: initial denaturation at 94°C for 5 min; 34 cycles of denaturation at 94°C for 30 s, annealing at 45.3°C or 53.7°C (as applicable) for 30 s, and extension at 72°C for 30 s, followed by a final extension at 72°C for 10 min. Subsequently, PCR products were electrophoresed for 20 min at 130 V and 180 W. Following electrophoresis, the DNA bands were analyzed to assess their quality (Figure 1). The qualified samples were sent to Shanghai Sangon Biotech Co., Ltd. (Shanghai, China) for sequencing.

### 2.7. Sequence Alignment and Genotyping

Sequence alignment and analysis were conducted using DNAMAN sequence analysis software (Version 5.2.2). Only variants confirmed by bidirectional sequencing and with a Phred quality score of ≥ Q30 were retained. Genotyping was performed manually using the Chromas Chromatogram Viewer (Version 2.4.1). A heterozygous state was assigned only when the height of the secondary peak reached at least 35% of that of the main peak.

Samples with poor chromatogram quality or sequencing failures were recorded as missing data. Samples exhibiting a genotyping loss rate exceeding 5% were excluded from subsequent association analyses. To validate the reliability of the workflow, 10% of the samples were randomly selected for repeat testing (from PCR to sequencing), and the final genotype concordance rate was 100%.

### 2.8. Statistical Analysis

Genotype and allele frequencies, polymorphism information content (PIC), effective number of alleles (Ne), and expected heterozygosity (He) were calculated using Microsoft Excel 2021 software. Statistical analyses were performed using SPSS software (Version 27.0.1). One‐way analysis of variance (ANOVA) was conducted to assess the associations of the *LCORL* and *LTBP2* genes with growth performance and slaughter traits in Charolais sheep, respectively. This study employed a fixed‐effects linear model to investigate the effects of genotype, age group, and sex on growth traits in Charolais sheep. The basic form of the model is as follows:

(1) Yijkl=μ+gj+bk+sl+eijkl.

In the model, *Y*_*i* *j* *k* *l*_ represents the observed phenotypic value; *μ* is the population mean; *g*_*j*_ represents the effect of genotype or genetic effect; *b*_*k*_ denotes the effect of age; *s*_*l*_ indicates the effect of sex; and *e*_*i* *j* *k* *l*_ is the random residual error.

Multiple comparisons were analyzed by Duncan’s test. Differences were considered indicative of no significant difference at *p* value > 0.05, statistically significant at *p* value < 0.05 and highly significant at *p* value < 0.01. Significant and highly significant differences are designated with lowercase and uppercase letters, respectively. Data are presented as “mean ± standard error.” Pearson correlation matrix and variance inflation factor (VIF) were calculated for multicollinearity diagnosis, and four‐panel residual diagnostic plots were generated to verify regression assumptions and identify influential outliers using Python 3.14; model optimization was realized by backward tracing from high‐*R* ^2^ equations to eliminate redundant explanatory variables.

## 3. Results

### 3.1. Sequencing Analysis of SNP Locus

**FIGURE 2:**
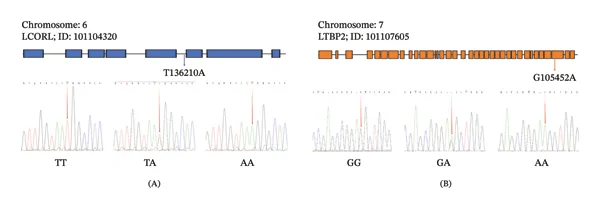
Identification of SNPs in the *LCORL* (A) and *LTBP2* (B) genes using Chromas Chromatogram Viewer.

Comparative analyses of the *LCORL* and *LTBP2* genes, performed repeatedly, identified a SNP (T136210A) in *LCORL* and detected another (G105452A) in *LTBP2* (Figure 2).

### 3.2. Genetic Diversity of *LCORL* and *LTBP2* Genes

Genetic diversity at two SNP loci, T136210A (*LCORL*) and G105452A (*LTBP2*), was analyzed in Charolais sheep (Table 2). The T136210A locus exhibited moderate polymorphism, with PIC values ranging from 0.25 to 0.5. Expected He ranged from 0.47 to 0.50, and the Ne varied between 1.89 and 2.00, indicating that this locus maintains considerable genetic diversity within the adult sheep population, with the highest diversity observed among rams. In contrast, the analysis of the lamb population revealed lower genetic diversity at this locus, although it remained higher than that of the G105452A locus. The G105452A locus displayed low polymorphism, with PIC values between 0 and 0.24. Corresponding values of He (0.00–0.28) and Ne (1.00–1.38) further indicated reduced genetic diversity at this locus.

**TABLE 2:** Analysis of genetic diversity at the *LCORL* and *LTBP2* loci in Charolais sheep.

Gene	Gene loci	Gender	Genotype frequency			Allele frequency		PIC	He	Ne	*χ* ^2^	*p*
			MM	Mm	mm	M	m					
*LCORL*	T136210A	Ewe	0.38	0.48	0.14	0.62	0.38	0.36	0.47	1.89	0.003883136	0.950312131
		Ram	0.22	0.56	0.22	0.50	0.50	0.38	0.50	2.00	0.111111111	0.73888268
		Ewe lamb	0.63	0.37	0	0.81	0.19	0.26	0.30	1.43	1.394628099	0.237625077
		Ram lamb	0.56	0.44	0	0.78	0.22	0.29	0.35	1.53	2.204081633	0.137645846
		Slaughtered ewe	0.60	0.20	0.20	0.70	0.30	0.33	0.42	1.72	1.371882086	0.241488778
		Slaughtered ram	0.60	0.20	0.20	0.70	0.30	0.33	0.42	1.72	1.371882086	0.241488778

*LTBP2*	G105452A	Ewe	0.75	0.23	0.02	0.86	0.14	0.21	0.24	1.31	0.054170514	0.815958958
		Ram	0.85	0.15	0	0.92	0.08	0.13	0.14	1.17	0.090277778	0.763824315
		Ewe lamb	0.67	0.33	0	0.83	0.17	0.24	0.28	1.38	0.720	0.396143909
		Ram lamb	0.89	0.11	0	0.94	0.06	0.10	0.10	1.12	0.093425606	0.759866559
		Slaughtered ewe	1.0	0	0	1.0	0	0	0	1.00	0	0
		Slaughtered ram	1.0	0	0	1.0	0	0	0	1.00	0	0

### 3.3. Gene Substitution Effect Analysis of *LCORL* and *LTBP2* Genes

The negative additive effect value of *LCORL* at the T136210A locus in Charolais sheep suggests a positive influence of this gene on production performance. Similarly, the negative additive effect value of *LTBP2* at the G105452A locus indicates its beneficial role in enhancing production traits in this breed (Table 3).

**TABLE 3:** Analysis of gene substitution effects at two loci within the *LCORL* and *LTBP2* genes.

Gene	Gene loci	Gender	Dominant effect	Additive effect	B gene average effect	A gene average effect	The average effect of B instead of A
			*d*	*a*	*a*1	*a*2	
*LCORL*	T136210A	Ewe	9.00	−5.00	−1.77	1.09	−2.86
		Ram	3.00	0	0	0	0
		Ewe lamb	1.50	−8.50	−6.16	1.40	−7.56
		Ram lamb	4.50	−7.50	−3.89	1.11	−5.00
		Slaughtered ewe	−1.00	−1.00	−0.98	0.42	−1.40
		Slaughtered ram	−1.00	−1.00	−0.98	0.42	−1.40

*LTBP2*	G105452A	Ewe	−7.00	−16.00	−18.21	2.88	−21.09
		Ram	−3.50	−5.50	−7.81	0.65	−8.46
		Ewe lamb	0	−6.00	−5.00	1.00	−6.00
		Ram lamb	−9.00	−12.00	−18.89	1.11	−20.00
		Slaughtered ewe	−5.00	−5.00	−10.00	0	−10.00
		Slaughtered ram	−5.00	−5.00	−10.00	0	−10.00

### 3.4. Analysis of Growth Performance of *LCORL* and *LTBP2* Genes

The Charolais sheep population was categorized into four distinct groups according to age and sex. A correlation analysis was then performed to assess 16 growth performance traits within each group.

At the T136210A locus of *LCORL* in adult ewes, the TT and TA genotypes exhibited significant advantages over AA in BaH, WH, SH, FW, WAW, and HW (*p* < 0.01), along with BH, CaC, CD, and CW (*p* < 0.05). In contrast, the AA genotype was associated with significantly greater CC and BL compared to TT and TA (*p* < 0.05). No significant differences were detected between TT and TA. At the G105452A locus of *LTBP2*, the AA genotype outperformed both GA and GG across all measured traits (*p* < 0.05).

In adult rams, at the T136210A locus of *LCORL*, the AA genotype showed significant advantages over TT in BL, CD, and CaC (*p* < 0.01) and was also significantly superior in BH, WH, SH, FW, CD, CW, and WAW (*p* < 0.05). In contrast, the TT genotype was associated with significantly greater HH, LL, and HW, while TA outperformed AA in BaH, LL, and HW. No significant differences were detected at the G105452A locus of *LTBP2*. No significant effect on growth performance was observed at the T136210A locus of *LCORL* in lambs. At the G105452A locus of *LTBP2*, ewe lambs with the GG genotype exhibited significantly greater BW, CC, LiL, and HW than those with GA (*p* < 0.05), with HW showing a highly significant difference (*p* < 0.01). No significant differences were observed in ram lambs at this locus (Table 4).

**TABLE 4:** Growth performance in relation to *LCORL* and *LTBP2* genotypes.

Gene	Gene loci	Gender	Genotype	BW (kg)	BH (cm)	BaH (cm)	WH (cm)	SH (cm)	HH (cm)	FW (cm)	CaC (cm)	CC (cm)	LiL (cm)	LL (cm)	BL (cm)	CD (cm)	CW (cm)	WAW (cm)	HW (cm)
*LCORL*	T136210A	Ewe	TT (176/462)	81.34 ± 1.18	72.00 ± 0.23	71.34 ± 0.26^aA^	71.91 ± 0.27^aA^	71.72 ± 0.30^aA^	65.81 ± 0.47^abAB^	15.59 ± 0.06^aA^	9.68 ± 0.04^aA^	123.56 ± 0.88^b^	21.59 ± 0.16	45.06 ± 0.48	62.34 ± 0.29^bB^	36.52 ± 0.13^aA^	35.83 ± 0.30^aA^	34.02 ± 0.41^aA^	34.62 ± 0.24^aA^
			TA (220/462)	78.63 ± 0.95	72.23 ± 0.24	72.13 ± 0.23^aA^	72.56 ± 0.25^aA^	72.46 ± 0.28^aA^	67.05 ± 0.34^aA^	15.38 ± 0.05^aA^	9.47 ± 0.05^bAB^	124.85 ± 0.72^ab^	21.55 ± 0.13	45.60 ± 0.45	63.15 ± 0.33^bB^	36.75 ± 0.17^aA^	36.20 ± 0.20^aA^	32.68 ± 0.39^aAB^	34.89 ± 0.21^aA^
			AA (66/462)	78.00 ± 1.31	72.08 ± 0.42	69.58 ± 0.24^bB^	69.80 ± 0.46^bB^	70.17 ± 0.56^bB^	65.17 ± 0.44^bB^	14.53 ± 0.22^bB^	9.37 ± 0.06^bB^	126.59 ± 0.66^a^	21.00 ± 0.52	45.00 ± 1.03	66.50 ± 0.40^aA^	35.00 ± 0.23^bB^	33.23 ± 0.39^bB^	30.87 ± 0.55^bB^	31.72 ± 0.35^bB^
		Ram	TT (4/18)	82.25 ± 6.78	68.25 ± 0.14^b^	70.50 ± 1.44^ab^	71.10 ± 1.21^b^	69.75 ± 0.43^b^	66.00 ± 1.15^a^	15.50 ± 0.87^b^	10.50 ± 0.29^bB^	105.00 ± 4.62	24.25 ± 1.30	51.75 ± 1.30^b^	62.75 ± 0.14^bB^	31.00 ± 0.02^bB^	26.75 ± 0.14^b^	22.60 ± 2.66^b^	32.25 ± 0.72^aA^
			TA (10/18)	82.70 ± 2.06	70.26 ± 0.89^a^	71.80 ± 1.14^a^	72.96 ± 0.89^ab^	73.24 ± 0.80^a^	67.96 ± 0.71^a^	16.30 ± 0.33^ab^	11.00 ± 0.29^bAB^	105.50 ± 1.30	23.90 ± 0.62	54.00 ± 0.87^b^	65.00 ± 0.82^bB^	34.88 ± 0.43^aA^	29.04 ± 0.67^a^	22.20 ± 1.06^b^	30.42 ± 0.63^bAB^
			AA (4/18)	84.50 ± 2.02	70.00 ± 1.15^a^	68.25 ± 1.30^b^	73.75 ± 2.17^a^	74.00 ± 4.04^a^	61.75 ± 5.34^b^	16.75 ± 0.43^a^	11.75 ± 0.14^aA^	107.75 ± 1.01	25.00 ± 2.89	50.75 ± 0.14^a^	70.25 ± 3.03^aA^	34.75 ± 0.43^aA^	28.70 ± 0.17^a^	25.40 ± 1.50^a^	28.00 ± 1.15^cB^
		Ewe lamb	TT (51/81)	8.29 ± 0.20	42.88 ± 0.25	42.59 ± 0.31	43.21 ± 0.33	42.50 ± 0.30	37.32 ± 0.42	9.97 ± 0.11	7.90 ± 0.07	48.82 ± 0.42	15.71 ± 0.22	29.77 ± 0.99	29.59 ± 0.56	16.54 ± 0.15	12.28 ± 0.17	10.30 ± 0.17	13.91 ± 0.27
			TA (30/81)	7.65 ± 0.20	43.50 ± 0.28	42.60 ± 0.37	43.20 ± 0.44	43.15 ± 0.37	37.40 ± 0.71	10.30 ± 0.13	7.83 ± 0.17	47.40 ± 0.62	14.55 ± 0.21	31.40 ± 0.41	31.10 ± 0.91	15.57 ± 0.27	11.23 ± 0.16	9.40 ± 0.26	12.63 ± 0.42
		Ram lamb	TT (45/81)	9.40 ± 0.23	43.35 ± 0.32	43.18 ± 0.30	43.90 ± 0.29	43.33 ± 0.26	38.35 ± 0.36	10.27 ± 0.08	7.93 ± 0.14	49.73 ± 0.54	16.17 ± 0.30	32.00 ± 0.23	28.63 ± 0.42	15.99 ± 0.26	12.79 ± 0.24	11.20 ± 0.38	14.37 ± 0.38
			AT (36/81)	8.46 ± 0.19	42.625 ± 0.33	41.875 ± 0.34	42.85 ± 0.31	42.50 ± 0.39	38.08 ± 0.32	10.38 ± 0.09	7.63 ± 0.09	48.32 ± 0.46	15.67 ± 1.80	32.46 ± 1.38	30.00 ± 0.56	16.05 ± 0.32	11.96 ± 0.31	9.73 ± 0.27	13.67 ± 0.39

*LTBP2*	G105452A	Ewe	GG (330/440)	80.08 ± 0.62^bB^	71.21 ± 0.15^bB^	70.38 ± 0.17^cC^	70.85 ± 0.18^cC^	70.82 ± 0.18^bB^	64.47 ± 0.26^cB^	15.42 ± 0.04^bAB^	9.60 ± 0.03^bB^	127.06 ± 0.50^bB^	20.73 ± 0.16^bB^	44.65 ± 0.40^b^	64.56 ± 0.27^bB^	35.89 ± 0.10^bB^	35.55 ± 0.17^bB^	32.76 ± 0.41	34.06 ± 0.22^bB^
			GA (100/440)	77.50 ± 1.06^bB^	72.45 ± 0.17^bB^	72.70 ± 0.23^bB^	73.32 ± 0.46^bB^	70.90 ± 0.46^bB^	67.20 ± 0.49^bB^	14.97 ± 0.16^bB^	9.55 ± 0.09^bB^	124.75 ± 1.10^bB^	21.80 ± 0.18^abAB^	47.60 ± 0.58^ab^	62.90 ± 0.34^bB^	37.73 ± 0.28^aA^	33.69 ± 0.33^cC^	31.11 ± 0.80	35.20 ± 0.51^bB^
			AA (10/440)	111.00 ± 0.02^aA^	78.00 ± 0.03^aA^	76.00 ± 0.06^aA^	77.50 ± 0.01^aA^	78.50 ± 0.01^aA^	73.00 ± 0.03^aA^	16.00 ± 0.02^aA^	10.10 ± 0.03^aA^	144.00 ± 0.01^aA^	23.00 ± 0.03^aA^	49.00 ± 0.03^a^	69.00 ± 0.02^aA^	37.00 ± 0.03^aAB^	42.70 ± 0.04^aA^	33.00 ± 0.03	41.50 ± 0.01^aA^
		Ram	GG (11/13)	87.18 ± 3.49	71.21 ± 0.64	70.64 ± 0.82^a^	73.80 ± 0.97	74.34 ± 1.22	66.89 ± 1.12^a^	16.77 ± 0.26	11.27 ± 0.32	107.77 ± 1.79	25.18 ± 0.88	52.55 ± 1.25	68.09 ± 1.44	34.68 ± 0.55	28.32 ± 0.39	22.43 ± 1.03	28.86 ± 1.09
			GA (2/13)	82.00 ± 1.00	68.00 ± 0.02	66.00 ± 0.01^b^	70.25 ± 0.25	69.00 ± 2.00	58.25 ± 5.75^b^	15.75 ± 0.25	11.75 ± 0.25	106.75 ± 0.75	22.25 ± 2.25	48.50 ± 2.50	66.50 ± 1.50	34.85 ± 0.85	29.25 ± 0.25	25.65 ± 2.35	28.75 ± 1.25
		Ewe lamb	GG (48/72)	8.42 ± 0.19^a^	42.88 ± 0.24	42.50 ± 0.35	43.58 ± 0.27	42.92 ± 0.27	37.54 ± 0.47	10.25 ± 0.97	8.13 ± 0.09	49.33 ± 0.47^a^	16.17 ± 0.29^a^	29.00 ± 1.21	30.63 ± 0.48	16.62 ± 0.19^a^	12.18 ± 0.16^aA^	9.91 ± 0.20	13.08 ± 0.32
			GA (24/72)	7.42 ± 0.07^b^	43.17 ± 0.28	42.83 ± 0.31	43.17 ± 0.47	42.33 ± 0.35	35.92 ± 0.54	10.33 ± 0.14	7.68 ± 0.11	46.33 ± 0.35^b^	14.25 ± 0.26^b^	33.17 ± 0.37	29.00 ± 0.24	15.42 ± 0.22^b^	10.92 ± 0.16^bB^	9.42 ± 0.24	13.58 ± 0.27
		Ram lamb	GG (72/81)	8.75 ± 0.21	42.68 ± 0.27	42.13 ± 0.29	42.90 ± 0.28	42.65 ± 0.28	38.18 ± 0.26	10.25 ± 0.07	7.73 ± 0.10	48.50 ± 0.44	15.94 ± 0.18	31.83 ± 0.28	29.25 ± 0.41	15.90 ± 0.22	12.30 ± 0.22	10.28 ± 0.28	13.74 ± 0.30
			GA (9/81)	8.50 ± 0.14	42.00 ± 0.52	42.83 ± 0.30	43.57 ± 0.49	42.83 ± 0.85	39.00 ± 0.50	10.00 ± 0.14	7.50 ± 0.14	49.27 ± 0.94	16.83 ± 0.22	31.83 ± 0.65	27.83 ± 0.79	16.73 ± 0.41	12.73 ± 0.41	11.50 ± 0.38	14.63 ± 0.77

*Note:* Different lowercase superscript letters denote the significant differences at *p* < 0.05, and uppercase superscript letters indicate the significant differences at *p* < 0.01.

Abbreviations: BaH, back height; BH, body height; BL, body length; BW, body weight; CaC, cannon circumference; CC, chest circumference; CD, chest depth; CW, chest width; FW, forehead width; HH, hip height; HW, hip width; LiL, limb length; LL, leg length; SH, sacral height; WAW, waist angle width; WH, waist height.

### 3.5. Analysis of Slaughter Performance of *LCORL* and *LTBP2* Genes

At the T136210A locus of *LCORL* in slaughtered ewes, the TA genotype exhibited significant differences from AA in PLW, EMA, GR value, and DP (*p* < 0.01). The TT genotype also differed significantly from AA in PLW, and from TA in BoW (*p* < 0.01), and demonstrated significant differences in BT compared to both TA and AA (*p* < 0.05). At the G105452A locus of *LTBP2*, only the GG genotype was detected.

At the T136210A locus of *LCORL* in slaughtered rams, the AA genotype exhibited significant advantages over both TT and TA in PLW, GR value, and BT, and over TA in DP (*p* < 0.01). The TT genotype showed significant superiority to TA and AA in CaW, to AA in NMP (*p* < 0.01), and a significant advantage over AA in BoW (*p* < 0.05). At the G105452A locus of *LTBP2*, only the GG genotype was observed (Table 5).

**TABLE 5:** Slaughter performance in relation to *LCORL* and *LTBP2* genotypes.

Gene	Gene loci	Gender	Genotype	PLW (kg)	CaW (kg)	BoW (kg)	DP (%)	NMP (%)	EMA (cm^2^)	GR value (mm)	BT (mm)
*LCORL*	T136210A	Slaughtered ewe	TT (6/10)	61.00 ± 0.66^aA^	33.40 ± 0.79^a^	4.62 ± 0.19^aA^	54.75 ± 1.13^abAB^	47.20 ± 1.14	32.63 ± 1.54^bA^	16.53 ± 0.49^aA^	7.00 ± 0.37^bB^
			TA (2/10)	61.00 ± 0.03^aA^	33.000 ± 0.04^ab^	4.26 ± 0.02^cB^	54.10 ± 0.02^bB^	47.12 ± 0.01	33.80 ± 0.01^aA^	10.68 ± 0.03^bB^	8.00 ± 0.02^aA^
			AA (2/10)	59.50 ± 0.02^bB^	32.80 ± 0.03^b^	4.50 ± 0.01^bA^	55.13 ± 0.02^aA^	47.56 ± 0.02	29.00 ± 0.01^cB^	16.69 ± 0.01^aA^	8.00 ± 0.02^aA^
		Slaughtered ram	TT (6/10)	68.50 ± 2.22^aA^	38.87 ± 1.59^aA^	5.43 ± 0.16^a^	56.70 ± 1.02^aAB^	48.74 ± 1.27^aA^	31.40 ± 1.74^cB^	16.49 ± 0.27^aA^	7.33 ± 0.21^aA^
			TA (2/10)	65.50 ± 0.02^bB^	36.60 ± 0.01^bB^	5.40 ± 0.01^a^	55.88 ± 0.01^bB^	47.63 ± 0.01^bB^	34.30 ± 0.02^bA^	14.41 ± 0.01^bB^	7.00 ± 0.03^bB^
			AA (2/10)	62.50 ± 0.02^cC^	35.80 ± 0.03^bB^	5.30 ± 0.02^b^	57.28 ± 0.02^aA^	48.80 ± 0.04^aA^	35.50 ± 0.02^aA^	13.99 ± 0.01^cC^	6.00 ± 0.02^cC^

*LTBP2*	G105452A	Slaughtered ewe	GG (10/10)	63.90 ± 2.42	35.76 ± 1.17	5.06 ± 0.31	56.01 ± 0.53	48.11 ± 0.61	33.96 ± 1.33	16.00 ± 0.76	7.20 ± 0.37
		Slaughtered ram	GG (10/10)	63.50 ± 1.36	35.24 ± 1.34	4.86 ± 0.14	55.34 ± 0.93	47.67 ± 0.96	30.98 ± 1.09	14.96 ± 0.74	7.20 ± 0.25

*Note:* Different lowercase superscript letters denote the significant differences at *p* < 0.05, and uppercase superscript letters indicate the significant differences at *p* < 0.01.

Abbreviations: BoW, bone weight; BT, backfat thickness; CaW, carcass weight; DP, dressing percentage; EMA, eye muscle area; NMP, net meat percentage; PLW, preslaughter live weight.

### 3.6. Analysis of Meat Quality Performance of *LCORL* and *LTBP2* Genes

In Charolais sheep, the T136210A locus of the *LCORL* gene significantly affects meat quality traits. In slaughtered ewes, the TT genotype was associated with significantly higher dry matter, cooked meat rate, meat color (*L* ^∗^ and *b* ^∗^ values), and pH, along with lower protein content and reduced drip loss compared to AA. In slaughtered rams, the AA genotype exhibited significantly greater dry matter, protein content, and meat color (*L* ^∗^ and *b* ^∗^ values) than both TT and TA, whereas the TT genotype showed significantly higher shear force and pH compared to TA and AA. In contrast, for the G105451A locus of the *LTBP2* gene, no significant effect of genotype was observed on any of the measured meat quality indicators in the studied population (Table 6).

**TABLE 6:** Meat quality performance in relation to *LCORL* and *LTBP2* genotypes.

Gene	Gene loci	Gender	Genotype	Dry matter (%)	Protein content (%)	Fat content (%)	Ash content (%)	Drip loss (%)	Cooked meat rate (%)	Meat color			Shear force (N)	pH
										*L*	*a*	*b*		
*LCORL*	T136210A	Slaughtered ewe	TT (6/10)	28.30 ± 1.24^aA^	21.83 ± 1.37^bB^	5.37 ± 0.51	1.17 ± 0.24	5.17 ± 1.75^bB^	74.64 ± 2.62^bB^	31.61 ± 0.65^aA^	8.64 ± 2.06^ab^	5.40 ± 0.84^aAB^	63.57 ± 1.69^a^	6.09 ± 0.04^aA^
			TA (2/10)	27.60 ± 0.09^abAB^	21.10 ± 0.08^bB^	5.20 ± 0.04	1.10 ± 0.04	5.68 ± 0.06^bAB^	77.38 ± 0.07^aAB^	31.86 ± 0.14^aA^	9.74 ± 0.05^a^	5.71 ± 0.03^aA^	62.05 ± 0.11^b^	6.12 ± 0.06^aA^
			AA (2/10)	26.80 ± 0.12^bB^	23.60 ± 0.10^aA^	5.30 ± 0.07	1.00 ± 0.06	7.33 ± 0.05^aA^	78.98 ± 0.03^aA^	30.09 ± 0.12^bB^	7.75 ± 0.02^b^	4.55 ± 0.06^bB^	63.31 ± 0.09^ab^	6.00 ± 0.03^bB^
		Slaughtered ram	TT (6/10)	29.43 ± 0.59^bB^	20.47 ± 0.70^bB^	7.63 ± 1.99^aA^	1.10 ± 0.08^bB^	3.42 ± 1.00	69.07 ± 4.72	33.60 ± 0.49^bB^	9.25 ± 1.29	5.82 ± 0.51^bB^	63.14 ± 0.26^aA^	6.14 ± 0.04^aA^
			TA (2/10)	29.40 ± 0.05^bB^	21.80 ± 0.36^aAB^	4.50 ± 0.33^bB^	1.10 ± 0.06^bB^	3.95 ± 0.12	69.77 ± 0.17	31.58 ± 0.15^cC^	9.29 ± 0.10	5.76 ± 0.08^bB^	60.25 ± 0.07^cC^	6.04 ± 0.01^bB^
			AA (2/10)	32.40 ± 0.07^aA^	22.40 ± 0.12^aA^	7.00 ± 0.27^aA^	1.20 ± 0.05^aA^	4.18 ± 0.32	66.92 ± 0.21	37.18 ± 0.22^aA^	8.63 ± 0.27	7.65 ± 0.07^aA^	61.19 ± 0.10^bB^	6.05 ± 0.03^bB^

*LTBP2*	G105451A	Slaughtered ewe	GG (10/10)	27.86 ± 1.13	22.04 ± 1.34	5.32 ± 0.40	1.12 ± 0.20	5.70 ± 1.59	76.05 ± 2.72	31.36 ± 0.82	8.68 ± 1.70	5.29 ± 0.76	63.22 ± 1.43	6.08 ± 0.05
		Slaughtered ram	GG (10/10)	30.02 ± 3.11	21.12 ± 1.47	6.88 ± 1.96	1.12 ± 0.08	3.68 ± 0.84	68.78 ± 3.74	33.91 ± 1.88	9.13 ± 1.02	6.18 ± 0.85	62.17 ± 1.26	6.10 ± 0.06

*Note:* Different lowercase superscript letters denote the significant differences at *p* < 0.05, and uppercase superscript letters indicate the significant differences at *p* < 0.01.

### 3.7. Sensitivity Analysis of Genotype–Phenotype Association

Sensitivity analyses based on permutation tests were performed separately for ram and ewe subgroups across all slaughtered performance (Figure 3) and meat quality traits (Figure 4). Notably, the overall conclusions derived from the original ANOVA were completely consistent with those obtained from permutation tests for all measured traits. This consistency fully verifies the robustness and reliability of the statistical results in the present study, indicating that the conclusions were not biased by the unbalanced sample distribution among the three genotypes (TT, TA, and AA).

FIGURE 3Slaughter performance permutation sensitivity test. (A) corresponds to slaughtered ewe, and (B) corresponds to slaughtered ram.

**Figure:**
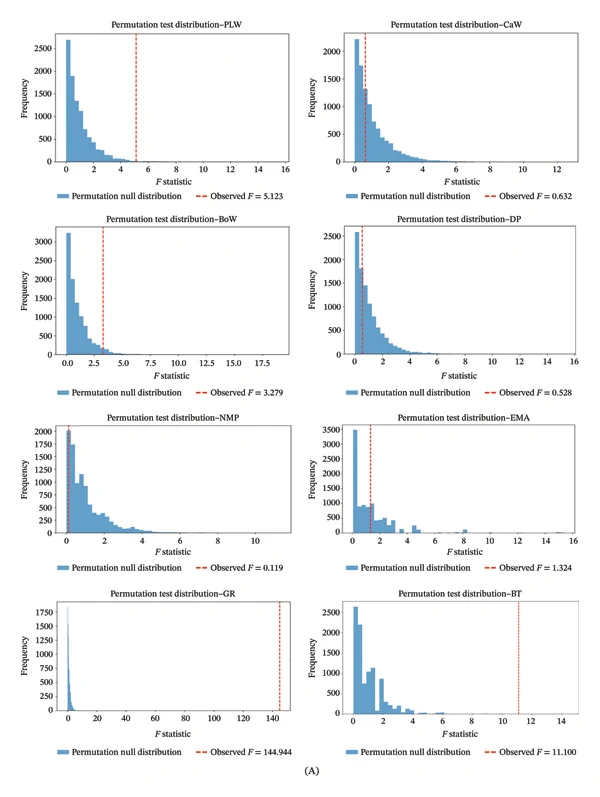

**Figure:**
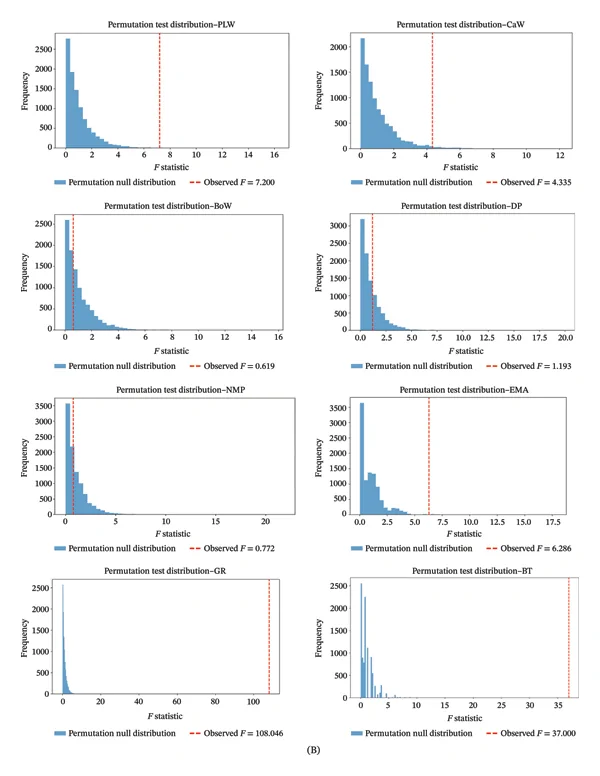

FIGURE 4Meat quality performance permutation sensitivity test. (A) corresponds to slaughtered ewe, and (B) corresponds to slaughtered ram.

**Figure:**
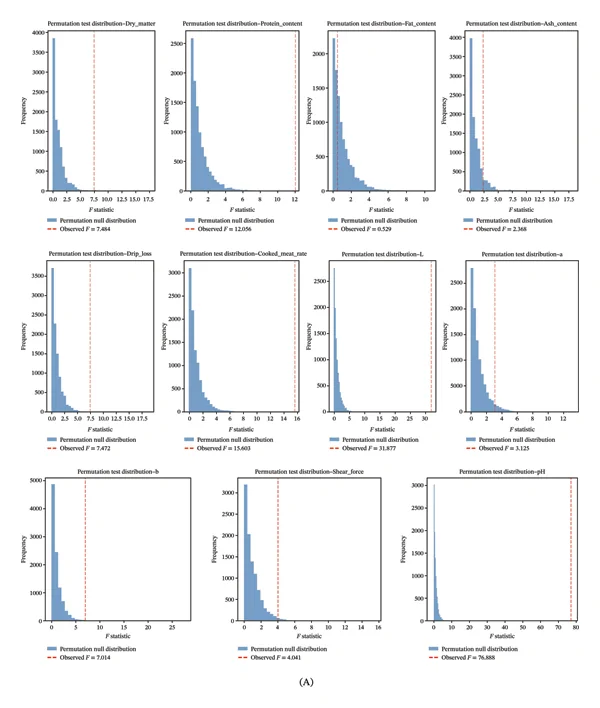

**Figure:**
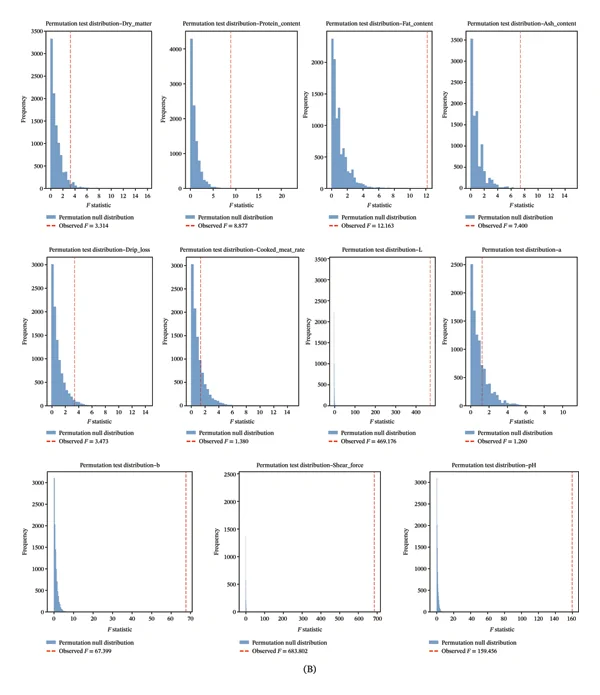

### 3.8. Correlation Analysis of BW and Growth Performance

In adult ewes, BW exhibited extremely significant correlations with BH, BaH, WH, SH, HH, CC, LL, CD, CW, and HW; significant correlations were also observed with FW, CaC, BL, and WAW. In adult rams, BW showed extremely significant correlations with CaC, CC, and BL, while significant correlations were found with BH, WH, FW, and CD (Table 7).

**TABLE 7:** Correlation coefficients between body weight and growth performance traits in adult Charolais sheep.

Ram	Ewe															
	BW	BH	BaH	WH	SH	HH	FW	CaC	CC	LiL	LL	BL	CD	CW	WAW	HW
BW	—	0.463^∗∗^	0.292^∗∗^	0.437^∗∗^	0.371^∗∗^	0.233^∗∗^	0.164^∗∗^	0.330^∗∗^	0.721^∗∗^	0.006	0.269^∗∗^	0.152^∗∗^	0.294^∗∗^	0.517^∗∗^	0.177^∗∗^	0.613^∗∗^
BH	0.463^∗^	—	0.819^∗∗^	0.747^∗∗^	0.647^∗∗^	0.638^∗∗^	−0.115^∗^	−0.126^∗∗^	0.347^∗∗^	0.437^∗∗^	0.318^∗∗^	0.076	0.336^∗∗^	0.293^∗∗^	0.197^∗∗^	0.486^∗∗^
BaH	0.311	0.742^∗∗^	—	0.772^∗∗^	0.668^∗∗^	0.643^∗∗^	−0.107^∗^	−0.116^∗^	0.253^∗∗^	0.452^∗∗^	0.348^∗∗^	−0.054	0.318^∗∗^	0.301^∗∗^	0.311^∗∗^	0.364^∗∗^
WH	0.461^∗^	0.681^∗∗^	0.682^∗∗^	—	0.767^∗∗^	0.656^∗∗^	−0.182^∗∗^	0.018	0.218^∗∗^	0.330^∗∗^	0.412^∗∗^	0.010	0.396^∗∗^	0.208^∗∗^	0.143^∗∗^	0.307^∗∗^
SH	0.301	0.612^∗∗^	0.496^∗∗^	0.921^∗∗^	—	0.541^∗∗^	0.153^∗∗^	−0.072	0.205^∗∗^	0.219^∗∗^	0.304^∗∗^	−0.061	0.308^∗∗^	0.419^∗∗^	0.383^∗∗^	0.368^∗∗^
HH	0.040	0.601^∗∗^	0.499^∗∗^	0.550^∗∗^	0.698^∗∗^	—	−0.235^∗∗^	−0.246^∗∗^	0.156^∗∗^	0.581^∗∗^	0.058	0.079	0.371^∗∗^	0.268^∗∗^	0.277^∗∗^	0.165^∗∗^
FW	0.433^∗^	0.604^∗∗^	0.342	0.574^∗∗^	0.584^∗∗^	0.352	—	0.343^∗∗^	0.125^∗∗^	−0.183^∗∗^	−0.081	0.042	0.149^∗∗^	0.282^∗∗^	0.086	0.358^∗∗^
CaC	0.657^∗∗^	0.598^∗∗^	0.149	0.303	0.172	0.027	0.376	—	0.427^∗∗^	−0.327^∗∗^	−0.090	0.350^∗∗^	0.152^∗∗^	0.004	−0.077	0.172^∗∗^
CC	0.920^∗∗^	0.540^∗∗^	0.268	0.499^∗∗^	0.384	−0.002	0.472^∗^	0.708^∗∗^	—	−0.144^∗∗^	−0.051	0.304^∗∗^	0.217^∗∗^	0.440^∗∗^	0.246^∗∗^	0.514^∗∗^
LiL	0.096	0.392^∗^	0.143	0.565^∗∗^	0.776^∗∗^	0.752^∗∗^	0.213	0.125	0.120	—	0.155^∗∗^	−0.157^∗∗^	0.095^∗^	−0.069	0.011	0.059
LL	0.038	0.040	0.551^∗∗^	0.303	0.105	−0.183	0.080	−0.394^∗^	−0.003	−0.306	—	−0.139^∗∗^	−0.078	−0.048	−0.278^∗∗^	0.199^∗∗^
BL	0.505^∗∗^	0.432^∗^	0.212	0.719^∗∗^	0.784^∗∗^	0.476^∗^	0.624^∗∗^	0.412^∗^	0.544^∗∗^	0.579^∗∗^	−0.252	—	−0.095^∗^	0.191^∗∗^	−0.233^∗∗^	0.053
CD	0.390^∗^	0.682^∗∗^	0.361	0.420^∗^	0.390^∗^	0.325	0.070	0.708^∗∗^	0.559^∗∗^	0.383	−0.331	0.404^∗^	—	0.362^∗∗^	0.302^∗∗^	0.258^∗∗^
CW	0.320	−0.204	−0.027	0.208	0.094	−0.440^∗^	−0.312	0.008	0.425^∗^	−0.086	0.363	0.055	0.204	—	0.535^∗∗^	0.598^∗∗^
WAW	0.143	0.156	0.182	0.005	−0.140	−0.386	−0.322	0.378	0.288	−0.160	0.085	−0.080	0.590^∗∗^	0.472^∗^	—	0.195^∗∗^
HW	0.081	−0.372	0.172	−0.262	−0.443^∗^	−0.486^∗^	−0.428^∗^	−0.341	−0.084	−0.516^∗∗^	0.604^∗∗^	−0.431^∗^	−0.297	0.407^∗^	0.382	—

*Note:* The upper triangle presents the correlation coefficients between ewe weight and growth performance traits, while the lower triangle displays those between ram weight and growth performance traits. Data marked with ^∗∗^ indicate a highly significant correlation (*p* < 0.01), ^∗^ indicates a significant correlation (*p* < 0.05), and the absence of an asterisk denotes no significant correlation (*p* > 0.05).

Abbreviations: BaH, back height; BH, body height; BL, body length; BW, body weight; CaC, cannon circumference; CC, chest circumference; CD, chest depth; CW, chest width; FW, forehead width; HH, hip height; HW, hip width; LiL, limb length; LL, leg length; SH, sacral height; WAW, waist angle width; WH, waist height.

BH, BaH, WH, SH, FW, CC, LiL, CD, CW, and HW exhibited highly significant correlations with BW in ewe lambs, while BL showed a significant correlation. In ram lambs, highly significant correlations with BW were observed for BH, BaH, WH, SH, HH, CaC, CC, LL, CD, CW, WAW, and HW, with LiL demonstrating a significant correlation (Table 8).

**TABLE 8:** Correlation coefficients between body weight and growth performance traits in Charolais lambs.

Ram lamb	Ewe lamb															
	BW	BH	BaH	WH	SH	HH	FW	CaC	CC	LiL	LL	BL	CD	CW	WAW	HW
BW	—	0.485^∗∗^	0.435^∗∗^	0.676^∗∗^	0.575^∗∗^	0.152	0.577^∗∗^	0.164	0.883^∗∗^	0.501^∗∗^	0.106	0.227^∗^	0.303^∗∗^	0.443^∗∗^	0.081	0.316^∗∗^
BH	0.513^∗∗^	—	0.835^∗∗^	0.688^∗∗^	0.583^∗∗^	0.073	0.463^∗∗^	−0.036	0.519^∗∗^	−0.001	0.132	0.279^∗^	−0.205	0.110	−0.044	−0.020
BaH	0.507^∗∗^	0.833^∗∗^	—	0.620^∗∗^	0.435^∗∗^	0.075	0.462^∗∗^	−0.031	0.535^∗∗^	0.185	0.045	0.144	−0.087	0.143	−0.128	0.035
WH	0.346^∗∗^	0.743^∗∗^	0.847^∗∗^	—	0.767^∗∗^	0.123	0.654^∗∗^	0.157	0.678^∗∗^	0.306^∗∗^	0.195	0.243^∗^	0.144	0.124	−0.108	0.030
SH	0.458^∗∗^	0.530^∗∗^	0.692^∗∗^	0.689^∗∗^	—	0.487^∗∗^	0.572^∗∗^	0.169	0.552^∗∗^	0.309^∗∗^	0.046	0.567^∗∗^	0.228^∗^	0.013	−0.315^∗∗^	−0.287^∗∗^
HH	0.467^∗∗^	0.328^∗∗^	0.349^∗∗^	0.354^∗∗^	0.508^∗∗^	—	−0.009	−0.020	0.278^∗^	0.357^∗∗^	0.118	0.267^∗^	0.458^∗∗^	0.286^∗∗^	−0.241^∗^	−0.190
FW	0.147	0.398^∗∗^	0.141	0.328^∗∗^	0.225^∗^	0.188	—	0.323^∗∗^	0.469^∗∗^	0.120	0.179	0.255^∗^	0.000	−0.108	−0.369^∗∗^	−0.095
CaC	0.614^∗∗^	0.222^∗^	0.314^∗∗^	0.244^∗^	0.462^∗∗^	0.084	0.042	—	0.160	0.346^∗∗^	0.116	0.046	0.515^∗∗^	0.149	0.174	0.067
CC	0.805^∗∗^	0.577^∗∗^	0.585^∗∗^	0.368^∗∗^	0.452^∗∗^	0.446^∗∗^	0.248^∗^	0.509^∗∗^	—	0.670^∗∗^	0.190	0.064	0.372^∗∗^	0.553^∗∗^	0.054	0.289^∗∗^
LiL	0.249^∗^	0.174	0.341^∗∗^	0.277^∗^	0.543^∗∗^	0.516^∗∗^	−0.130	0.309^∗∗^	0.315^∗∗^	—	0.146	−0.066	0.507^∗∗^	0.422^∗∗^	−0.067	0.117
LL	0.325^∗∗^	0.632^∗∗^	0.527^∗∗^	0.563^∗∗^	0.384^∗∗^	0.234^∗^	0.589^∗∗^	0.240^∗^	0.342^∗∗^	−0.039	—	−0.284^∗^	−0.117	−0.131	−0.163	0.160
BL	0.103	−0.032	−0.152	−0.114	0.259^∗^	0.034	0.113	0.432^∗∗^	−0.005	0.378^∗∗^	−0.130	—	0.030	−0.300^∗∗^	−0.340^∗∗^	−0.518^∗∗^
CD	0.511^∗∗^	0.283^∗^	0.496^∗∗^	0.181	0.378^∗∗^	0.220^∗^	−0.327^∗∗^	0.416^∗∗^	0.582^∗∗^	0.223^∗^	0.070	−0.110	—	0.534^∗∗^	0.307^∗∗^	0.193
CW	0.703^∗∗^	0.484^∗∗^	0.475^∗∗^	0.194	0.433^∗∗^	0.520^∗∗^	−0.043	0.364^∗∗^	0.765^∗∗^	0.304^∗∗^	0.212	−0.065	0.633^∗∗^	—	0.504^∗∗^	0.558^∗∗^
WAW	0.629^∗∗^	0.273^∗^	0.419^∗∗^	0.127	0.140	0.231^∗^	−0.240^∗^	0.274^∗^	0.608^∗∗^	0.029	0.162	−0.420^∗∗^	0.570^∗∗^	0.672^∗∗^	—	0.733^∗∗^
HW	0.713^∗∗^	0.488^∗∗^	0.399^∗∗^	0.123	0.108	0.319^∗∗^	0.026	0.188	0.692^∗∗^	−0.070	0.325^∗∗^	−0.268^∗^	0.460^∗∗^	0.653^∗∗^	0.821^∗∗^	—

*Note:* The upper triangle presents the correlation coefficients between ewe lamb weight and growth performance traits, while the lower triangle displays those between ram lamb weight and growth performance traits. Data marked with ^∗∗^ indicate a highly significant correlation (*p* < 0.01), ^∗^ indicates a significant correlation (*p* < 0.05), and the absence of an asterisk denotes no significant correlation (*p* > 0.05).

Abbreviations: BaH, back height; BH, body height; BL, body length; BW, body weight; CaC, cannon circumference; CC, chest circumference; CD, chest depth; CW, chest width; FW, forehead width; HH, hip height; HW, hip width; LiL, limb length; LL, leg length; SH, sacral height; WAW, waist angle width; WH, waist height.

### 3.9. Path Analysis of BW and Growth Performance

The BaH of adult ewes exhibited the greatest direct effect on BW (−0.571), followed by CC (0.513), WH (0.492), CW (0.392), BH (0.389), LL (0.252), SH (−0.250), BL (−0.198), CaC (0.155), FW (0.119), CD (−0.093), HH (0.084), HW (−0.025), and WAW (0.002). Regarding indirect effects, BaH also had the largest influence (0.734), followed by BL (0.516), LiL (0.374), CW (0.276), HH (0.207), LL (0.195), SH (−0.167), BH (−0.105), WH (−0.101), CD (0.069), CC (0.047), WAW (−0.044), CaC (0.043), FW (0.028), and HW (−0.020). CD exhibited the strongest direct negative effect on adult ram BW (−1.858), followed by WH (−1.771), BH (1.315), CaC (1.099), FW (−1.007), BL (0.976), HH (0.929), CW (0.681), BaH (0.615), and CC (0.485). Regarding indirect effects, WH had the greatest influence (1.991), followed by CD (1.977), FW (1.211), BH (−1.114), HH (−0.815), CaC (−0.785), BL (−0.736), CW (−0.567), CC (0.435), and BaH (−0.434) (Table 9).

**TABLE 9:** Path coefficients of growth performance traits on body weight in adult Charolais sheep.

Sex	Independent variable	Correlation coefficient	Direct effect (path coefficient)	Indirect effect (indirect path coefficient)												
				BH	BaH	WH	SH	HH	FW	CaC	LL	BL	CD	CW	WAW	HW
Ewe	BH	0.463	0.389	—	−0.468	0.368	−0.162	0.054	−0.014	−0.020	0.080	−0.015	−0.031	0.115	0.000	−0.012
	BaH	0.292	−0.571	0.319	—	0.380	−0.167	0.054	−0.013	−0.018	0.088	0.011	−0.030	0.118	0.001	−0.009
	WH	0.437	0.492	0.291	−0.441	—	−0.192	0.055	−0.022	0.003	0.104	−0.002	−0.037	0.082	0.000	−0.008
	SH	0.371	−0.250	0.252	−0.381	0.377	—	0.045	0.018	−0.011	0.077	0.012	−0.029	0.164	0.001	−0.009
	HH	0.233	0.084	0.248	−0.367	0.323	−0.135	—	−0.028	−0.038	0.015	−0.016	−0.035	0.105	0.001	−0.004
	FW	0.164	0.119	−0.045	0.061	−0.090	−0.038	−0.020	—	0.053	−0.021	−0.008	−0.014	0.111	0.000	−0.009
	CaC	0.330	0.155	−0.049	0.066	0.009	0.018	−0.021	0.041	—	−0.023	−0.069	−0.014	0.002	0.000	−0.004
	CC	0.721	0.513	0.135	−0.144	0.107	−0.051	0.013	0.015	0.066	−0.013	−0.060	−0.020	0.172	0.000	−0.013
	LiL	0.006	—	0.170	−0.258	0.162	−0.055	0.049	−0.022	−0.051	0.039	0.031	−0.009	−0.027	0.000	−0.001
	LL	0.269	0.252	0.124	−0.199	0.203	−0.076	0.005	−0.010	−0.014	—	0.028	0.007	−0.019	−0.001	−0.005
	BL	0.152	−0.198	0.030	0.031	0.005	0.015	0.007	0.005	0.054	−0.035	—	0.009	0.075	0.000	−0.001
	CD	0.294	−0.093	0.131	−0.182	0.195	−0.077	0.031	0.018	0.024	−0.020	0.019	—	0.142	0.001	−0.006
	CW	0.517	0.392	0.114	−0.172	0.102	−0.105	0.023	0.034	0.001	−0.012	−0.038	−0.034	—	0.001	−0.015
	WAW	0.177	0.002	0.077	−0.178	0.070	−0.096	0.023	0.010	−0.012	−0.070	0.046	−0.028	0.210	—	−0.005
	HW	0.613	−0.025	0.189	−0.208	0.151	−0.092	0.014	0.043	0.027	0.050	−0.010	−0.024	0.234	0.000	—

Ram	BH	0.463	1.315	—	0.456	−1.205	0.569	—	−0.608	0.658	—	0.422	−1.267	−0.139	—	—
	BaH	0.311	0.615	0.975	—	−1.208	0.461	—	−0.344	0.164	—	0.207	−0.670	−0.019	—	—
	WH	0.461	−1.770	0.895	0.420	—	0.856	—	−0.577	0.334	—	0.702	−0.781	0.142	—	—
	SH	0.301	0.929	0.805	0.305	−1.631	—	—	−0.588	0.189	—	0.765	−0.724	0.064	—	—
	FW	0.433	−1.007	0.794	0.210	−1.015	0.543	—	—	0.414	—	0.609	−0.131	−0.213	—	—
	CaC	0.657	1.099	0.787	0.092	−0.537	0.160	—	−0.379	—	—	0.402	−1.316	0.006	—	—
	CC	0.920	0.485	0.710	0.165	−0.883	0.357	—	−0.475	0.779	—	0.531	−1.039	0.290	—	—
	BL	0.505	0.976	0.568	0.131	−1.274	0.728	—	−0.628	0.453	—	—	−0.751	0.037	—	—
	CD	0.390	−1.858	0.896	0.222	−0.744	0.362	—	−0.071	0.778	—	0.395	—	0.139	—	—
	CW	0.320	0.681	−0.268	−0.017	−0.368	0.088	—	0.314	0.009	—	0.054	−0.379	—	—	—

Abbreviations: BaH, back height; BH, body height; BL, body length; CaC, cannon circumference; CD, chest depth; CW, chest width; FW, forehead width; HH, hip height; HW, hip width; LL, leg length; SH, sacral height; WAW, waist angle width; WH, waist height.

CC exhibited the greatest direct effect on BW (0.594) in ewe lambs, followed by BaH (0.416), CW (0.363), FW (0.305), LiL (0.297), HH (−0.282), BH (−0.195), BL (−0.175), WH (−0.160), CD (−0.104), CaC (0.059), SH (−0.056), and HW (0.041). Regarding indirect effects, CD had the strongest indirect influence (0.699), followed by WH (0.674), HH (0.667), SH (0.564), BH (0.557), BL (0.474), CaC (0.451), HW (0.398), LiL (0.375), CW (0.348), CC (0.335), FW (0.144), and BaH (0.009). Ram lamb HW (0.518) exhibited the strongest direct effect on BW, followed by CaC (0.406), loin height (0.329), CW (0.166), LL (−0.162), HH (0.123), BH (−0.114), FW (0.113), CC (0.111), WAW (−0.083), BaH (−0.040), CD (−0.028), LiL (−0.024), and SH (−0.021). In contrast, CC showed the largest indirect effect on weight (0.728), followed by BH (0.706), WAW (0.700), BaH (0.633), LL (0.599), CW (0.583), SH (0.562), HH (0.552), CD (0.529), LiL (0.368), CaC (0.232), HW (0.173), WH (0.164), and FW (0.162) (Table 10).

**TABLE 10:** Path coefficients of growth performance traits on body weight in Charolais lambs.

Sex	Independent variable	Correlation coefficient	Direct effect (path coefficient)	Indirect effect (indirect path coefficient)														
				BH	BaH	WH	SH	HH	FW	CaC	CC	LiL	LL	BL	CD	CW	WAW	HW
Ewe lamb	BH	0.361	−0.195	—	0.354	−0.112	−0.034	0.007	0.153	0.015	0.251	−0.005	—	−0.076	0.009	−0.005	—	0.000
	BaH	0.424	0.416	−0.166	—	−0.108	−0.036	−0.056	0.160	0.004	0.287	0.034	—	−0.103	−0.005	0.000	—	−0.002
	WH	0.513	−0.16	−0.137	0.281	—	−0.047	−0.078	0.138	0.011	0.395	0.119	—	−0.032	−0.024	0.045	—	0.003
	SH	0.507	−0.056	−0.119	0.267	−0.134	—	−0.120	0.186	0.022	0.378	0.170	—	−0.075	−0.024	0.023	—	−0.010
	HH	0.384	−0.282	0.005	0.082	−0.044	−0.024	—	0.069	0.009	0.319	0.186	—	−0.013	−0.058	0.136	—	0.000
	FW	0.448	0.305	−0.098	0.218	−0.072	−0.034	−0.063	—	0.017	0.233	0.038	—	−0.052	0.006	−0.040	—	−0.009
	CaC	0.51	0.059	−0.050	0.025	−0.029	−0.021	−0.044	0.087	—	0.270	0.169	—	−0.029	−0.042	0.113	—	0.002
	CW	0.927	0.594	−0.083	0.201	−0.106	−0.036	−0.151	0.120	0.027	—	0.223	—	−0.057	−0.066	0.248	—	0.015
	LiL	0.671	0.297	0.003	0.048	−0.064	−0.032	−0.176	0.039	0.034	0.446	—	—	−0.047	−0.071	0.188	—	0.007
	BL	0.299	−0.175	−0.085	0.245	−0.029	−0.024	−0.021	0.090	0.010	0.193	0.080	—	—	−0.001	0.029	—	−0.013
	CD	0.592	−0.104	0.017	0.021	−0.036	−0.013	−0.156	−0.016	0.024	0.379	0.202	—	−0.001	—	0.266	—	0.012
	CW	0.711	0.363	0.002	0.000	−0.020	−0.004	−0.106	−0.033	0.018	0.406	0.154	—	−0.014	−0.076	—	—	0.021
	HW	0.438	0.041	0.000	−0.016	−0.011	0.014	−0.002	−0.066	0.004	0.212	0.054	—	0.054	−0.031	0.186	—	—

Ram lamb	BH	0.591	−0.114	—	−0.034	0.259	−0.014	0.076	0.049	0.120	0.075	−0.009	−0.104	—	−0.008	0.098	−0.025	0.223
	BaH	0.592	−0.040	−0.098	—	0.288	−0.016	0.072	0.026	0.159	0.075	−0.012	−0.092	—	−0.013	0.092	−0.036	0.188
	WH	0.492	0.329	−0.090	−0.035	—	−0.016	0.077	0.046	0.137	0.058	−0.011	−0.098	—	−0.005	0.054	−0.014	0.061
	SH	0.541	−0.021	−0.074	−0.031	0.255	—	0.079	0.038	0.211	0.062	−0.015	−0.075	—	−0.009	0.077	−0.015	0.059
	HH	0.675	0.123	−0.071	−0.023	0.205	−0.014	—	0.050	0.115	0.072	−0.010	−0.078	—	−0.007	0.098	−0.027	0.242
	FW	0.275	0.113	−0.049	−0.009	0.135	−0.007	0.055	—	0.043	0.040	−0.001	−0.101	—	0.007	0.016	0.016	0.017
	CaC	0.639	0.406	−0.034	−0.016	0.111	−0.011	0.035	0.012	—	0.060	−0.011	−0.049	—	−0.012	0.069	−0.024	0.102
	CC	0.838	0.111	−0.077	−0.027	0.171	−0.012	0.080	0.040	0.221	—	−0.010	−0.072	—	−0.015	0.130	−0.050	0.349
	LiL	0.343	−0.024	−0.045	−0.020	0.147	−0.013	0.054	0.003	0.178	0.047	—	−0.016	—	−0.005	0.048	−0.006	−0.004
	LL	0.437	−0.162	−0.073	−0.023	0.198	‐0.010	0.059	0.070	0.123	0.050	−0.002	—	—	−0.004	0.057	−0.016	0.170
	CD	0.500	−0.028	−0.032	−0.019	0.061	−0.007	0.030	−0.029	0.170	0.061	−0.005	−0.020	—	—	0.106	−0.048	0.261
	CW	0.747	0.166	−0.067	−0.022	0.106	−0.010	0.073	0.011	0.168	0.087	−0.007	−0.056	—	−0.018	—	−0.058	0.376
	WAW	0.616	−0.083	−0.034	−0.017	0.057	−0.004	0.040	−0.021	0.120	0.067	−0.002	−0.032	—	−0.016	0.116	—	0.426
	HW	0.691	0.518	−0.049	−0.015	0.038	−0.002	0.057	0.004	0.080	0.075	0.000	−0.053	—	−0.014	0.120	−0.068	—

Abbreviations: BaH, back height; BH, body height; BL, body length; CaC, cannon circumference; CC, chest circumference; CD, chest depth; CW, chest width; FW, forehead width; HH, hip height; HW, hip width; LiL, limb length; LL, leg length; SH, sacral height; WAW, waist angle width; WH, waist height.

### 3.10. Stepwise Multiple Regression Analysis Results of BW and Growth Performance

Prior to establishing population‐specific stepwise multiple linear regression equations, residual diagnostic validation was prespecified to assess model statistical assumptions and fitting robustness for ewe, ram, ewe lamb, and ram lamb (Figure 5). Residual‐versus‐fitted value exhibited randomly scattered residuals around the zero baseline, satisfying the homoscedasticity assumption. Normal Q–Q plots and frequency distribution histograms of residuals verified that residuals approximately followed a normal distribution across all four cohorts, meeting the fundamental prerequisites for linear regression analysis. Using 4/N as the Cook’s distance threshold to screen influential observations, no extreme outliers that substantially distort overall regression trends were identified in any group. These diagnostic outcomes guaranteed the rationality of subsequent regression modeling, excluded potential bias from abnormal samples, and prevented artificial inflation of the coefficient of determination (*R* ^2^).

FIGURE 5Residual diagnosis for stepwise regression models constructed from four populations (A–D). Each panel contains four subplots: residual versus fitted value plot, normal Q–Q plot of residuals, frequency distribution histogram of residuals, and Cook’s distance plot for extreme sample identification. (A–D) represent the ewe, ram, ewe lamb, and ram lamb, respectively. The red dashed line marks Cook’s distance threshold of 4/N. Residuals of all four models approximately followed normal distribution with homoscedasticity, satisfying linear regression assumptions. No severely influential outliers distorting overall regression trends were observed, verifying reliable model fitting for each population.

**Figure:**
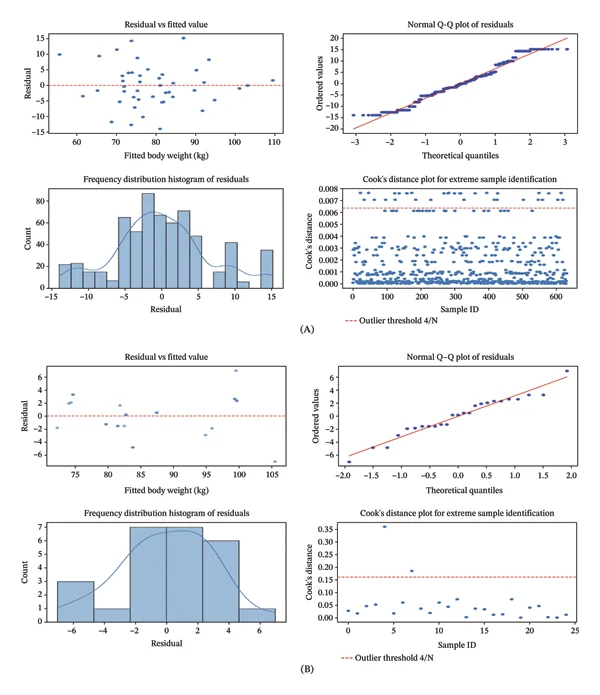

**Figure:**
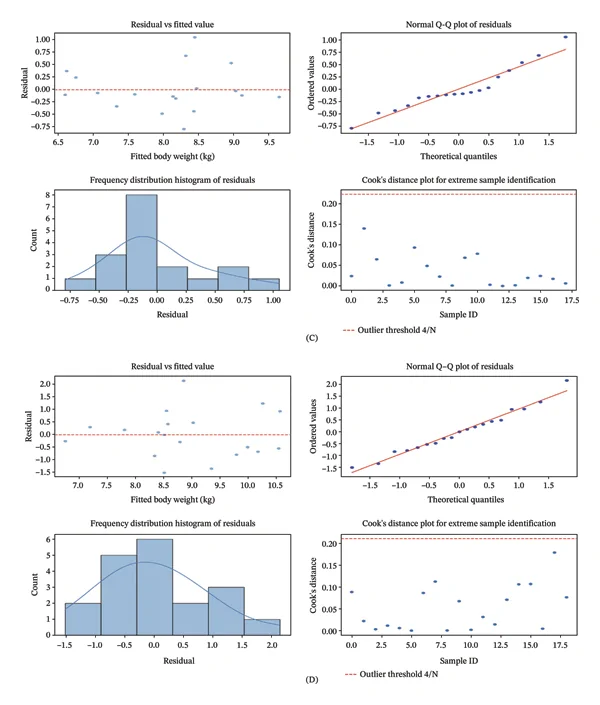

The optimal regression equations for BW were derived as follows: For ewes, the equation is BW = 0.690CC + 0.522LL + 1.546CW + 1.863WH − 2.386BaH + 1.613BH − 0.610BL + 3.422CaC − 0.819SH + 1.555FW − 0.575CD + 0.227HH − 118.193, and for rams, BW = 1.472CC + 0.509HW − 88.303. The coefficients of determination (*R* ^2^) for these multiple regression models were 0.794 and 0.961, respectively. This indicates that 79.4% of the variance in adult ewe weight can be explained by CC, LL, CW, WH, BaH, BH, BL, CaC, SH, FW, CD, and HH. Similarly, 96.1% of the variance in adult ram weight is accounted for by CC and HW. These high *R* ^2^ values demonstrate that both models are well‐constructed (Table 11).

**TABLE 11:** Stepwise multiple regression analysis of the effect of growth performance on body weight in adult Charolais sheep.

Sex	Model	*R* ^2^	Adjusted *R* ^2^	Standardized estimated error	*F*‐value	*p* value	VIF
Ewe	BW = 0.978CC − 42.283	0.519	0.518	9.934	497.334	< 0.001	< 5
	BW = 0.999CC + 0.645LL − 74.169	0.613	0.612	8.922	363.917	< 0.001	< 5
	BW = 0.846CC + 0.659LL + 1.049CW − 93.032	0.667	0.665	8.291	305.511	< 0.001	< 5
	BW = 0.809CC + 0.517LL + 0.951CW + 0.606WH − 122.065	0.686	0.683	8.063	249.080	< 0.001	< 5
	BW = 0.829CC + 0.566LL + 1.154CW + 1.584WH − 1.520BaH − 95.750	0.735	0.732	7.414	252.585	< 0.001	< 5
	BW = 0.781CC + 0.557LL + 1.152CW + 1.325WH − 2.114BaH + 1.070BH − 105.367	0.752	0.748	7.180	229.649	< 0.001	< 5
	BW = 0.816CC + 0.534LL + 1.196CW + 1.364WH − 2.278BaH + 1.172BH − 87.624	0.761	0.758	7.049	206.761	< 0.001	< 5
	BW = 0.697CC + 0.536LL + 1.326CW + 1.155WH − 2.263BaH + 1.524BH − 0.426BL + 3.586CaC − 115.075	0.777	0.773	6.824	196.962	< 0.001	< 5
	BW = 0.682CC + 0.537LL + 1.509CW + 1.508WH − 2.255BaH + 1.596BH − 0.514BL + 3.592CaC − 0.499SH − 111.789	0.784	0.779	6.726	181.841	< 0.001	< 5
	BW = 0.704CC + 0.526LL + 1.422CW + 1.790WH − 2.252BaH + 1.548BH − 0.489BL + 2.524CaC − 0.700SH + 1.264FW − 124.266	0.788	0.783	6.664	167.622	< 0.001	< 5
	BW = 0.693CC + 0.482LL + 1.541CW + 1.992WH − 2.300BaH + 1.635BH − 0.562BL + 2.849CaC − 0.800SH + 1.453FW − 0.488CD − 118.992	0.791	0.786	6.620	155.055	< 0.001	< 5
	BW = 0.690CC + 0.522LL + 1.546CW + 1.863WH − 2.386BaH + 1.613BH − 0.610BL + 3.422CaC − 0.819SH + 1.555FW − 0.575CD + 0.227HH − 118.193	0.794	0.788	6.586	144.122	< 0.001	< 5

Ram	BW = 1.457CC − 71.329	0.937	0.936	1.888	1569.300	< 0.001	< 5
	BW = 1.472CC + 0.509HW − 88.303	0.961	0.961	1.484	1303.783	< 0.001	< 5

Abbreviations: BaH, back height; BH, body height; BL, body length; BW, body weight; CaC, cannon circumference; CC, chest circumference; CD, chest depth; CW, chest width; FW, forehead width; HH, hip height; LL, leg length; SH, sacral height; WH, waist height.

The optimal regression equations for BW were derived as follows: for ewe lambs, BW = 0.690CC + 0.522LL + 1.546CW + 1.863WH − 2.386BaH + 1.613BH − 0.610BL + 3.422CaC − 0.819SH + 1.555FW − 0.575CD + 0.227HH − 118.193, and for ram lambs, BW = 0.095CC + 0.770CaC + 0.251HW + 0.136HH − 10.381. The *R* ^2^ values were 0.909 and 0.819, respectively. This indicates that 90.9% of the variability in ewe lamb BW can be explained by CC, LL, CW, WH, BaH, BH, BL, CaC, SH, FW, CD, and HH. Similarly, 81.9% of the variability in ram lamb BW can be accounted for by CC, CaC, HW, and HH. Both models demonstrate a strong fit and are well‐constructed (Table 12).

**TABLE 12:** Stepwise multiple regression analysis of the effect of growth performance on body weight in Charolais lambs.

Sex	Model	*R* ^2^	Adjusted *R* ^2^	Standardized estimated error	*F*‐value	*p* value	VIF
Ewe lamb	BW = 0.370CC − 9.817	0.780	0.778	0.635	471.098	< 0.001	< 5
	BW = 0.329CC − 0.370FW − 11.568	0.814	0.811	0.586	288.661	< 0.001	< 5
	BW = 0.311CC + 0.499FW + 0.143WAW − 13.452	0.831	0.827	0.561	214.020	< 0.001	< 5
	BW = 0.310CC + 0.454FW + 0.195WAW + 0.055BL − 15.129	0.859	0.854	0.514	197.686	< 0.001	< 5
	BW = 0.287CC + 0.392FW + 0.027WAW + 0.083BL + 0.160HW − 14.697	0.878	0.874	0.479	186.474	< 0.001	< 5
	BW = 0.287CC + 0.376FW + 0.084BL + 0.174HW − 14.472	0.878	0.874	0.478	234.316	< 0.001	< 5
	BW = 0.309CC + 0.430FW + 0.085BL + 0.170HW − 0.077BaH − 12.765	0.888	0.884	0.460	204.260	< 0.001	< 5
	BW = 0.312CC + 0.489FW + 0.086BL + 0.175HW − 0.091BaH − 0.156CaC − 11.768	0.893	0.888	0.450	178.662	< 0.001	< 5
	BW = 0.330CC + 0.447FW + 0.090BL + 0.161HW − 0.096BaH − 0.157CaC − 0.034HH − 10.688	0.899	0.893	0.441	161.012	< 0.001	< 5
	BW = 0.308CC + 0.509FW + 0.095BL + 0.140HW − 0.097BaH − 0.189CaC − 0.044HH + 0.113CW − 10.760	0.902	0.896	0.434	145.523	< 0.001	< 5
	BW = 0.289CC + 0.462FW + 0.085BL + 0.146HW − 0.103BaH − 0.201CaC − 0.059HH + 0.133CW + 0.072SH − 11.581	0.906	0.899	0.429	133.242	< 0.001	< 5
	BW = 0.243CC + 0.531FW + 0.092BL + 0.159BL − 0.098BaH − 0.279CaC − 0.069HH + 0.164CW + 0.081SH + 0.076LiL − 11.587	0.909	0.902	0.423	123.651	< 0.001	5–10

Ram lamb	BW = 0.358CC − 8.596	0.648	0.645	0.872	195.329	< 0.001	< 5
	BW = 0.296CC + 0.519CaC − 9.582	0.704	0.699	0.803	125.119	< 0.001	< 5
	BW = 0.147CC + 0.692CaC + 0.245HW − 7.088	0.788	0.782	0.684	128.587	< 0.001	< 5
	BW = 0.095CC + 0.770CaC + 0.251HW + 0.136HH − 10.381	0.819	0.812	0.634	116.795	< 0.001	< 5

Abbreviations: BaH, back height; BH, body height; BL, body length; BW, body weight; CaC, cannon circumference; CC, chest circumference; CD, chest depth; CW, chest width; FW, forehead width; HH, hip height; HW, hip width; LiL, limb length; LL, leg length; WH, waist height.

### 3.11. Correlation Analysis of BW and Slaughter Performance

The PLW, CaW, DP, NMP, EMA, GR value, and BT in slaughtered ewes were all highly significantly correlated with BW, while BoW showed a significant correlation with weight. In slaughtered rams, all slaughter traits exhibited highly significant correlations with BW (Table 13).

**TABLE 13:** Correlation coefficients between body weight and slaughter performance traits in Charolais sheep.

Slaughtered ram	Slaughtered ewe								
	BW	PLW	CaW	BoW	DP	NMP	EMA	GR value	BT
BW	—	−0.329^∗∗^	−0.347^∗∗^	−0.106^∗^	−0.204^∗∗^	−0.200^∗∗^	0.288^∗∗^	0.637^∗∗^	−0.164^∗∗^
PLW	0.194^∗∗^	—	0.502^∗∗^	0.829^∗∗^	−0.018	−0.180^∗∗^	−0.500^∗∗^	−0.165^∗∗^	−0.854^∗∗^
CaW	0.305^∗∗^	0.910^∗∗^	—	0.530^∗∗^	0.856^∗∗^	0.748^∗∗^	−0.187^∗∗^	0.322^∗∗^	−0.323^∗∗^
BoW	−0.293^∗∗^	0.372^∗∗^	−0.034	—	0.114^∗^	−0.111^∗^	−0.798^∗∗^	0.308^∗∗^	−0.899^∗∗^
DP	0.316^∗∗^	0.178^∗^	0.570^∗∗^	−0.807^∗∗^	—	0.974^∗∗^	0.088	0.469^∗∗^	0.138^∗∗^
NMP	0.347^∗∗^	0.321^∗∗^	0.685^∗∗^	−0.733^∗∗^	0.988^∗∗^	—	0.283^∗∗^	0.370^∗∗^	0.333^∗∗^
EMA	−0.624^∗∗^	0.469^∗∗^	0.501^∗∗^	0.148^∗^	0.286^∗∗^	0.322^∗∗^	—	−0.156^∗∗^	0.555^∗∗^
GR value	0.679^∗∗^	0.461^∗∗^	0.242^∗∗^	0.490^∗∗^	−0.357^∗∗^	−0.265^∗∗^	−0.494^∗∗^	—	−0.239^∗∗^
BT	0.645^∗∗^	−0.070	−0.166^∗^	0.000	−0.291^∗∗^	−0.254^∗∗^	−0.871^∗∗^	0.670^∗∗^	—

*Note:* The upper triangle presents the correlation coefficients between slaughtered ewe weight and slaughter performance traits, while the lower triangle displays those between slaughtered ram weight and slaughter performance traits. Data marked with ^∗∗^ indicate a highly significant correlation (*p* < 0.01), ^∗^ indicates a significant correlation (*p* < 0.05), and the absence of an asterisk denotes no significant correlation (*p* > 0.05).

Abbreviations: BoW, bone weight; BT, backfat thickness; BW, body weight; CaW, carcass weight; DP, dressing percentage; EMA, eye muscle area; NMP, net meat percentage; PLW, preslaughter live weight.

### 3.12. Path Analysis of BW and Slaughter Performance

For slaughtered ewes, the GR value exhibited the greatest direct effect on PLW (1.032), followed by DP (−0.720), EMA (0.594), and BT (−0.147). Regarding indirect effects, DP had the largest influence on PLW (0.516), followed by the GR value (−0.395), EMA (−0.306), and BT (−0.017). For slaughtered rams, the GR value also showed the largest direct effect on PLW (1.395), followed by BoW (−0.98), BT (−0.309), and CaW (−0.177). The largest indirect effect on PLW was observed for BT (0.964), followed by the GR value (−0.73), BoW (0.69), and CaW (0.421) (Table 14).

**TABLE 14:** Path coefficients of slaughter performance traits on body weight in Charolais sheep.

Sex	Independent variable	Correlation coefficient	Direct effect (path coefficient)	Indirect effect (indirect path coefficient)					
				CaW	BoW	DP	EMA	GR value	BT
Slaughtered ewe	DP	−0.204	−0.720	—	—	—	0.052	0.484	−0.020
	EMA	0.288	0.594	—	—	−0.063	—	−0.161	−0.082
	GR value	0.637	1.032	—	—	−0.338	−0.092	—	0.035
	BT	−0.164	−0.147	—	—	−0.100	0.330	−0.247	—

Slaughtered ram	CaW	0.305	−0.177	—	0.033	—	—	0.337	0.051
	BoW	−0.293	−0.980	0.006	—	—	—	0.684	0
	GR value	0.679	1.395	−0.043	−0.480	—	—	—	−0.207
	BT	0.645	−0.309	0.029	0	—	—	0.935	—

Abbreviations: BoW, bone weight; BT, backfat thickness; CaW, carcass weight; DP, dressing percentage; EMA, eye muscle area.

### 3.13. Correlation Analysis of BW and Meat Quality Performance

In slaughter ewes, dry matter content, drip loss, and shear force were highly significantly correlated with BW, while the ash content showed a significant correlation with BW. In slaughter rams, ash content, shear force, and pH were highly significantly correlated with BW (Table 15).

**TABLE 15:** Correlation coefficients between body weight and meat quality performance traits in Charolais sheep.

Slaughtered ram		Slaughtered ewe											
		BW (kg)	Dry matter (%)	Protein content (%)	Fat content (%)	Ash content (%)	Drip loss (%)	Cooked meat rate (%)	Meat color			Shear force (N)	pH
									*L*	*a*	*b*		
BW (kg)		—	0.568^∗∗^	−0.145	0.181	0.289^∗^	−0.588^∗∗^	−0.099	0.061	−0.273	−0.033	0.486^∗∗^	−0.053

Dry matter (%)		−0.359	—	−0.882^∗∗^	0.733^∗∗^	−0.285^∗^	−0.941^∗∗^	0.243	−0.014	−0.626^∗∗^	−0.337^∗^	−0.334^∗^	0.693^∗∗^

Protein content (%)		−0.216	−0.539^∗∗^	—	−0.654^∗∗^	0.340^∗^	0.858^∗∗^	−0.297^∗^	−0.121	0.457^∗∗^	0.213	0.564^∗∗^	−0.879^∗∗^

Fat content (%)		0.333	0.729^∗∗^	−0.800^∗∗^	—	−0.847^∗∗^	−0.488^∗∗^	0.391^∗∗^	−0.587^∗∗^	−0.939^∗∗^	−0.848^∗∗^	−0.763^∗∗^	0.560^∗∗^

Ash content (%)		−0.550^∗∗^	0.882^∗∗^	−0.448^∗^	0.571^∗∗^	—	0.014	−0.474^∗∗^	0.790^∗∗^	0.827^∗∗^	0.916^∗∗^	0.898^∗∗^	−0.324^∗^

Drip loss (%)		0.003	0.152	0.459^∗^	−0.091	−0.197	—	−0.281^∗^	−0.194	0.402^∗∗^	0.080	0.117	−0.601^∗∗^

Cooked meat rate (%)		0.095	−0.917^∗∗^	0.492^∗∗^	−0.756^∗∗^	−0.626^∗∗^	−0.437^∗^	—	−0.611^∗∗^	−0.564^∗∗^	−0.557^∗∗^	−0.365^∗∗^	−0.110

Meat color	*L*	0.017	0.273	0.353	0.239	0.338	0.328	−0.253	—	0.782^∗∗^	0.926^∗∗^	0.460^∗∗^	0.288^∗^
	*a*	0.089	−0.870^∗∗^	0.402^∗^	−0.684^∗∗^	−0.544^∗∗^	−0.548^∗∗^	0.991^∗∗^	−0.251	—	0.942^∗∗^	0.613^∗∗^	−0.254
	*b*	−0.346	−0.059	0.677^∗∗^	−0.299	0.216	0.109	0.221	0.802^∗∗^	0.218	—	0.662^∗∗^	−0.075

Shear force (N)		0.936^∗∗^	−0.291	−0.368^∗^	0.440^∗^	−0.344	−0.311	0.151	0.054	0.190	−0.265	—	−0.626^∗∗^

pH		0.929^∗∗^	−0.616^∗∗^	−0.087	0.086	−0.651^∗∗^	−0.246	0.440^∗^	−0.068	0.446^∗^	−0.227	0.929^∗∗^	—

*Note:* The upper triangle presents the correlation coefficients between slaughtered ewe weight and meat quality traits, while the lower triangle displays those between slaughtered ram weight and meat quality traits. Data marked with ^∗∗^ indicate a highly significant correlation (*p* < 0.01), ^∗^ indicates a significant correlation (*p* < 0.05), and the absence of an asterisk denotes no significant correlation (*p* > 0.05).

### 3.14. Path Analysis of BW and Meat Quality Performance

Table 16 indicates that the direct effect of dry matter on BW was greatest in slaughter ewes (1.679), followed by that on shear force (1.402), drip loss (0.835), and ash content (−0.504). Regarding indirect effects, dry matter exerted the strongest influence on BW (−1.110), followed by shear force (−0.916), ash content (0.792), and drip loss (−0.629).

**TABLE 16:** Path coefficients of meat quality performance traits on body weight in Charolais sheep.

Sex	Independent variable	Correlation coefficient	Direct effect (path coefficient)	Indirect effect (indirect path coefficient)				
				Dry matter (%)	Ash content (%)	Drip loss (%)	Shear force (N)	pH
Slaughtered ewe	Dry matter (%)	0.568	1.679	—	0.144	−0.786	−0.468	—
	Ash content (%)	0.289	−0.504	−0.478	—	0.011	1.259	—
	Drip loss (%)	−0.588	0.835	−0.786	−0.007	—	0.164	—
	Shear force (N)	0.486	1.402	−0.561	−0.453	0.098	—	—

Slaughtered ram	Ash content (%)	−0.550	−1.060	—	—	—	−0.876	1.386
	Shear force (N)	0.936	2.549	—	0.364	—	—	−1.978
	pH	0.929	−2.130	—	0.690	—	2.368	—

For slaughter rams, shear force exhibited the strongest direct effect on BW (2.549), followed by pH (−2.130) and ash content (−1.000). In terms of indirect effects, pH showed the greatest influence on BW (3.058), ahead of shear force (−1.614) and ash content (0.510).

### 3.15. Haplotype Combination Analysis of *LCORL* and *LTBP2* Genes

Analysis of SNP loci within the *LCORL* and *LTBP2* genes was performed using the SHEsis software platform (https://analysis.bio-x.cn/myAnalysis.php), which identified seven potential haplotype combinations (Table 17).

**TABLE 17:** Haplotype combinations of *LCORL* (T136210A) and *LTBP2* (G105452A) polymorphisms.

Haplotype	H1: TT	H2: TA	H3: AA
H1: GG	TTGG	TAGG	AAGG
H2: GA	TTGA	TAGA	AAGA
H3: AA	TTAA		

### 3.16. Correlation of Haplotype Combinations and Growth Performance

Seven haplotype combinations were identified in adult ewes. The TTAA haplotype combination exhibited significantly superior performance (*p* < 0.01) compared to other combinations across multiple traits, including BW, BH, BaH, WH, SH, HH, FW, CaC, CC, LL, BL, CW, and HW. The TAGA haplotype was significantly superior (*p* < 0.01) for CD and WAW, while the AAGG combination showed a significant advantage (*p* < 0.01) in BL. Additionally, the AAGA haplotype was significantly superior (*p* < 0.01) for LiL. In adult rams, four haplotype combinations were detected. The TAGA haplotype combination was significantly superior (*p* < 0.01) for CW and HW and significantly better (*p* < 0.05) for CaC and LL. The AAGG haplotype group outperformed others in BL and HW, whereas the TTGG group was significantly superior for WAW. Finally, the TAGG haplotype group exhibited a significant advantage in BH (Table 18).

**TABLE 18:** Haplotype combinations of two genes associated with growth performance in adult Charolais sheep.

Name	Haplotype	Sample size	BW (kg)	BH (cm)	BaH (cm)	WH (cm)	SH (cm)	HH (cm)	FW (cm)	CaC (cm)	CC (cm)	LiL (cm)	LL (cm)	BL (cm)	CD (cm)	CW (cm)	WAW (cm)	HW (cm)
Ewe	TTGG	121	83.41 ± 12.57^bB^	72.07 ± 1.83^cC^	71.36 ± 2.63^BcdC^	72.18 ± 2.48^bB^	71.73 ± 3.64^BbCD^	65.18 ± 6.56^cC^	15.55 ± 0.73^bAB^	9.71 ± 0.65^bB^	124.77 ± 10.86^cBC^	21.05 ± 1.70^cC^	44.50 ± 6.21^bB^	62.27 ± 3.38^cdCD^	36.47 ± 1.38^bcB^	35.76 ± 3.54^bcBC^	34.56 ± 5.57^abA^	34.67 ± 2.54^bB^
	TTGA	44	68.25 ± 11.70^eE^	70.25 ± 4.07^dDE^	70.13 ± 4.78^cdeCD^	69.75 ± 4.50^cC^	70.00 ± 3.58^dCD^	65.75 ± 4.87^cBC^	15.63 ± 0.97^bAB^	9.50 ± 0.36^bcdBC^	115.13 ± 6.36^dD^	22.75 ± 2.64^abAB^	45.63 ± 7.17^bAB^	60.88 ± 4.05^dD^	36.53 ± 2.75^bcB^	34.30 ± 3.67^dC^	32.80 ± 5.41^bcAB^	32.75 ± 2.97^cD^
	TTAA	22	111.00 ± 0.03^aA^	78.00 ± 0.02^aA^	76.00 ± 0.03^aA^	77.50 ± 0.04^aA^	78.50 ± 0.02^aA^	73.00 ± 0.03^aA^	16.00 ± 0.03^aA^	10.10 ± 0.01^aA^	144.00 ± 0.03^aA^	23.00 ± 0.02^aAB^	49.00 ± 0.03^aA^	69.00 ± 0.02^aA^	37.00 ± 0.03^bB^	42.70 ± 0.02^aA^	33.00 ± 0.03^bcAB^	41.50 ± 0.03^aA^
	TAGG	143	78.73 ± 9.50^bcBCD^	71.65 ± 3.25^cCD^	71.46 ± 3.21^cBC^	71.92 ± 3.39^bB^	72.02 ± 4.27^bBC^	65.85 ± 4.77^cBC^	15.35 ± 0.63^bB^	9.52 ± 0.56^bcBC^	125.12 ± 8.76^cBC^	21.23 ± 2.15^cC^	45.65 ± 6.77^bAB^	63.50 ± 5.49^bcBC^	35.95 ± 1.71^cB^	35.51 ± 2.26^bcdBC^	31.29 ± 5.73^cBC^	34.58 ± 2.94^bBC^
	TAGA	66	73.00 ± 16.27^deDE^	72.50 ± 3.38^cC^	72.92 ± 3.66^bB^	73.12 ± 3.88^bB^	72.42 ± 3.40^bB^	68.67 ± 4.79^bB^	15.33 ± 0.81^bB^	9.25 ± 0.91^cdC^	121.08 ± 11.97^cC^	22.00 ± 1.57^bcBC^	44.92 ± 6.87^bB^	61.42 ± 2.48^dCD^	38.43 ± 3.09^aA^	36.62 ± 3.10^bB^	35.62 ± 5.38^aA^	34.45 ± 2.58^bBC^
	AAGG	33	75.83 ± 10.16^cdCD^	69.33 ± 0.96^dE^	69.17 ± 2.13^eD^	67.93 ± 4.36^dD^	70.83 ± 2.94^bcBCD^	62.33 ± 1.73^dD^	15.50 ± 0.72^bAB^	9.50 ± 0.42^bcdBC^	129.33 ± 5.52^bB^	18.33 ± 4.57^dD^	44.00 ± 9.34^bB^	68.00 ± 2.49^aA^	33.67 ± 1.730^dC^	35.07 ± 1.66^cdBC^	33.00 ± 1.66^bcAB^	30.33 ± 0.48^dE^
	AAGA	33	80.17 ± 10.82^bcBC^	74.83 ± 2.70^bB^	70.00 ± 1.66^deCD^	71.67 ± 1.27^bB^	69.50 ± 5.76^dD^	68.00 ± 2.49^bBC^	13.57 ± 2.06^cC^	9.23 ± 0.53^dC^	123.83 ± 3.48^cC^	23.67 ± 0.96^aA^	46.00 ± 7.23^bAB^	65.00 ± 3.32^bB^	36.33 ± 0.48^bcB^	31.40 ± 3.25^eD^	28.73 ± 5.27^dC^	33.10 ± 3.47^cCD^

Ram	TTGG	4	82.25 ± 13.57	68.25 ± 0.29^ab^	70.50 ± 2.89	71.10 ± 2.43	69.75 ± 0.87	66.00 ± 2.31	15.50 ± 1.73	10.50 ± 0.58^c^	105.00 ± 9.24	24.25 ± 2.60	51.75 ± 2.60^b^	62.75 ± 0.29^b^	31.00 ± 0.02^bB^	26.75 ± 0.29^dC^	22.60 ± 5.31^ab^	32.25 ± 1.44^aA^
	TAGG	8	81.38 ± 6.67	71.08 ± 2.53^a^	71.75 ± 4.10	72.70 ± 3.13	72.55 ± 2.37	67.45 ± 2.24	16.13 ± 1.09	10.75 ± 0.89^bc^	104.88 ± 4.41	24.38 ± 1.90	53.50 ± 2.88^ab^	65.00 ± 2.93^ab^	34.60 ± 1.39^aA^	28.05 ± 0.39^cB^	23.25 ± 2.84^ab^	29.90 ± 1.89^abAB^
	TAGA	2	88.00 ± 0.03	67.00 ± 0.02^b^	72.00 ± 0.03	74.00 ± 0.04	76.00 ± 0.03	70.00 ± 0.03	17.00 ± 0.03	12.00 ± 0.01^a^	108.00 ± 0.02	22.00 ± 0.03	56.00 ± 0.03^a^	65.00 ± 0.01^ab^	36.00 ± 0.02^aA^	33.00 ± 0.02^aA^	18.00 ± 0.03^c^	32.50 ± 0.02^aA^
	AAGG	4	84.50 ± 4.04	70.00 ± 2.31^ab^	68.25 ± 2.60	73.75 ± 4.33	74.00 ± 8.08	61.75 ± 10.68	16.75 ± 0.87	11.75 ± 0.29^ab^	107.75 ± 2.02	25.00 ± 5.77	50.75 ± 0.29^b^	70.25 ± 6.06^a^	34.75 ± 0.87^aA^	28.70 ± 0.35^bB^	25.40 ± 3.00^a^	28.00 ± 2.31^bB^

*Note:* Different lowercase superscript letters denote the significant differences at *p* < 0.05, and uppercase superscript letters indicate the significant differences at *p* < 0.01.

Abbreviations: BaH, back height; BH, body height; BL, body length; BW, body weight; CaC, cannon circumference; CC, chest circumference; CD, chest depth; CW, chest width; FW, forehead width; HH, hip height; HW, hip width; LiL, limb length; LL, leg length; SH, sacral height; WAW, waist angle width; WH, waist height.

Four haplotype combinations were identified in ewe lambs. The TTGG combination was highly significantly superior for BW, CC, LiL, and CW. The TAGA combination was highly significantly superior in FW and significantly superior in LL. The TAGG combination was highly significantly superior in BL. The TTGA combination was significantly superior in HW. Four haplotype combinations were identified in ram lambs. The TAGA combination was highly significantly better for CD, CW, WAW, and HW and significantly superior in CC. The TTGA group was highly significantly superior in WH and SH and significantly superior in BaH and CaC (Table 19).

**TABLE 19:** Haplotype combinations of two genes associated with growth performance in Charolais lambs.

Name	Haplotype	Sample size	BW (kg)	BH (cm)	BaH (cm)	WH (cm)	SH (cm)	HH (cm)	FW (cm)	CaC (cm)	CC (cm)	LiL (cm)	LL (cm)	BL (cm)	CD (cm)	CW (cm)	WAW (cm)	HW (cm)
Ewe lamb	TTGG	33	8.86 ± 1.28^aA^	42.86 ± 1.89	42.41 ± 2.32	43.68 ± 2.10	43.23 ± 2.10^aA^	38.00 ± 2.79	10.18 ± 0.69^bB^	8.00 ± 0.48	50.09 ± 2.90^aA^	16.27 ± 1.46^aA^	28.86 ± 8.57^b^	30.50 ± 3.55^abAB^	16.79 ± 1.04^aA^	12.39 ± 1.21^aA^	10.24 ± 1.46^aA^	13.77 ± 2.19^ab^
	TTGA	18	7.25 ± 1.06^bB^	42.92 ± 1.69	42.92 ± 2.00	42.33 ± 2.62	41.17 ± 1.51^bB^	36.08 ± 3.01	9.58 ± 0.81^cB^	7.72 ± 0.57	46.50 ± 1.54^bB^	14.67 ± 1.18^bB^	31.42 ± 1.82^ab^	27.92 ± 4.27^bB^	16.08 ± 0.96^aA^	12.08 ± 1.24^abA^	10.42 ± 0.55^aA^	14.17 ± 1.48^a^
	TAGG	21	7.71 ± 1.31^bB^	43.57 ± 1.63	42.50 ± 2.31	42.93 ± 2.53	43.21 ± 2.27^aA^	37.86 ± 4.43	10.07 ± 0.69^bB^	7.86 ± 1.05	47.86 ± 3.82^bAB^	14.64 ± 1.41^bB^	30.07 ± 3.75^b^	32.29 ± 5.57^aA^	15.96 ± 1.59^aA^	11.54 ± 0.86^bA^	9.71 ± 1.40^aAB^	12.26 ± 2.66^b^
	TAGA	9	7.50 ± 0.43^bB^	43.33 ± 1.32	42.83 ± 1.25	43.83 ± 2.14	43.00 ± 1.50^aA^	36.33 ± 2.00	10.83 ± 0.25^aA^	7.77 ± 0.65	46.33 ± 1.80^bB^	14.33 ± 1.25^bB^	34.50 ± 0.75^a^	28.33 ± 0.50^bAB^	14.67 ± 0.25^bB^	10.50 ± 0.43^cB^	8.67 ± 1.25^bB^	13.50 ± 0.87^ab^

Ram lamb	TTGG	13	9.50 ± 1.68	43.33 ± 1.85	43.05 ± 2.14^ab^	43.69 ± 2.02^abAB^	43.15 ± 1.86^abAB^	38.48 ± 2.42	10.23 ± 0.56	7.92 ± 1.02^ab^	50.04 ± 3.81^ab^	16.23 ± 1.35	31.96 ± 2.80	28.42 ± 3.03^abAB^	16.12 ± 1.85^abAB^	13.09 ± 1.53^aAB^	11.31 ± 2.80^aAB^	14.45 ± 2.69^abAB^
	TTGA	2	8.75 ± 1.06	43.50 ± 4.95	44.00 ± 2.12^a^	45.25 ± 1.77^aA^	44.50 ± 0.71^aA^	37.50 ± 3.54	10.50 ± 0.71	8.00 ± 0.02^a^	47.75 ± 2.48^b^	15.75 ± 2.48	32.25 ± 3.89	30.000 ± 1.41^aA^	15.10 ± 0.14^bB^	10.85 ± 0.50^bC^	10.50 ± 0.71^abAB^	13.80 ± 3.11^bAB^
	TAGG	10	8.40 ± 1.29	42.55 ± 2.22	41.65 ± 2.20^b^	42.75 ± 1.93^bB^	42.65 ± 2.42^abAB^	38.00 ± 2.06	10.45 ± 0.55	7.70 ± 0.54^ab^	47.80 ± 2.71^b^	15.50 ± 1.99	32.35 ± 1.47	30.75 ± 3.32^aA^	15.76 ± 2.07^bAB^	11.65 ± 1.93^bBC^	9.22 ± 1.27^bB^	13.17 ± 2.32^bB^
	TAGA	2	8.75 ± 0.35	43.00 ± 0.71	43.00 ± 1.41^ab^	43.35 ± 2.33^bAB^	41.75 ± 3.18^bB^	38.50 ± 2.12	10.000 ± 0.71	7.25 ± 0.35^b^	50.90 ± 2.26^a^	16.50 ± 0.71	33.00 ± 1.41	26.25 ± 0.35^bB^	17.50 ± 0.71^aA^	13.50 ± 0.71^aA^	12.25 ± 0.35^aA^	16.15 ± 0.50^aA^

*Note:* Different lowercase superscript letters denote the significant differences at *p* < 0.05, and uppercase superscript letters indicate the significant differences at *p* < 0.01.

Abbreviations: BaH, back height; BH, body height; BL, body length; BW, body weight; CaC, cannon circumference; CC, chest circumference; CD, chest depth; CW, chest width; FW, forehead width; HH, hip height; HW, hip width; LiL, limb length; LL, leg length; SH, sacral height; WAW, waist angle width; WH, waist height.

## 4. Discussion

Charolais sheep are a globally recognized breed renowned for their meat production, characterized by rapid growth, early maturity, exceptional feed conversion efficiency, and strong adaptability. The Chao Mu meat sheep, developed in Liaoning Province by crossing Charolais rams with Small‐tailed Han ewes, represents the first new meat sheep breed independently bred in the region. Progeny sired by Charolais sheep exhibit superior growth performance compared to those from other breeds. Charolais sheep have played a significant role in enhancing the genetic progress and breeding of meat sheep in China. This study aims to analyze the influence of genotypes at two loci on 16 growth performance and eight slaughter traits in Charolais sheep. Additionally, the influence of combined haplotypes of these genes on growth traits was assessed. Existing research has identified multiple genes that influence body conformation traits in sheep, such as *PRKAA2* [18], *CD8B* [19], and *LRRC8B* [20]. The SNP site located on *LCORL* is designated as T136210A, while the corresponding SNP site on *LTBP2* is designated as G105452A. We further assessed population genetic diversity by calculating the PIC, expected He, and Ne. The PIC value greater than 0.5 indicates a high level of polymorphism, PIC values between 0.25 and 0.5 suggest a moderate degree of polymorphism, and PIC values below 0.25 correspond to low polymorphism. Higher He values reflect greater genetic diversity, while larger Ne values indicate an enhanced capacity to maintain alleles within populations, including mutant alleles. The PIC value of *LCORL* in Charolais sheep exhibited a moderate level of polymorphism, ranging from 0.25 to 0.5. The He value suggested abundant genetic diversity and substantial genetic variation at this locus. Furthermore, the high Ne value indicated a rich diversity of mutants within the population and stronger allelic stability. This gene locus in Charolais sheep may have been subject to mild, rather than strong, artificial selection. It exhibits high levels of genetic diversity and substantial potential for genetic variation, suggesting that greater genetic progress could be achieved in future breeding efforts. The PIC value of *LTBP2* in Charolais sheep was low, ranging from 0 to 0.24. The values of He (0.00–0.28) and Ne (1.00–1.38) further indicated limited genetic diversity at this locus. These results suggest that *LTBP2* may have been subjected to strong artificial selection, leading to complete fixation for homozygosity within the population of slaughtered lambs. Chi‐squared tests indicated that most populations were in Hardy–Weinberg equilibrium (*p* > 0.05), with the adult sheep population exhibiting complete conformity. A small number of loci exhibited allele fixation and deviated from Hardy–Weinberg equilibrium in this study, which was mainly attributed to long‐term intensive directional artificial selection [21]. Sustained selection for economic traits accumulates favorable alleles and eliminates inferior ones, ultimately resulting in homozygous fixation, a common genetic pattern in domesticated breeding populations [22]. Rechecking raw genotyping data ruled out false fixation caused by genotyping calling errors. These monomorphic fixed loci were excluded from the subsequent association analyses. Since the vast majority of markers conformed to Hardy–Weinberg equilibrium, our sample and genotyping data were validated to be reliable, and sporadic fixation represented a typical genomic signature driven by strong artificial selection [23].

Interpreting and analyzing the effects of gene substitutions revealed that mutations at these loci both exhibit negative additive effects and exert a positive influence on the trait. *LCORL* was associated with variations in growth performance. In ewes, the TT genotype conferred advantages in multiple traits (e.g., BaH, FW, and CW). In contrast, the AA genotype was associated with superior performance in CC and BL. In rams, the AA genotype demonstrated superiority in multiple traits (e.g., BL, CD, and HW). The TT genotype showed advantages in HH, LL, and WAW, while the TA genotype was associated with improved BaH, LL, and WAW. No clear advantage of any single genotype was observed in lambs. Differences in slaughter performance were also noted. In ewes, the TA genotype exhibited advantages in PLW, EMA, and GR value; the TT genotype showed benefits in PLW, BoW, and BT, whereas the AA genotype demonstrated a higher DP. In rams, the AA genotype was associated with superior EMA, GR value, and BT; the TT genotype displayed advantages in CaW, NMP, and BoW. Regarding *LTBP2*, the AA genotype was identified as favorable for growth performance in ewes, while no significant genotypic differences were observed in rams. In lambs, the GG genotype conferred advantages in ewe lamb BW, CC, LiL, and CW, although no significant differences were detected among genotypes in ram lambs. For slaughter traits, only the GG genotype was observed. Further analysis of these genes revealed distinct haplotype combinations. The most favorable haplotype for growth performance was TTAA in ewes, TAGA in rams, TTGG in ewe lambs, and TAGA in ram lambs. Stepwise multiple regression was adopted to screen key factors affecting sheep growth performance. Multicollinearity was evaluated using the correlation matrix and VIF values: Most variables had VIF below 5, and only a few ranged from 5 to 10, revealing merely mild collinearity originating from inherent genetic and physiological correlations among body measurements of meat‐type sheep. To solve excessively high fitting in several equations, we pruned redundant variables by backward tracing from high‐R^2^ models and further verified model rationality using residual diagnostic plots generated with Python 3.14. Residual analysis confirmed that all optimized models complied with the assumptions of ordinary least squares, delivering stable fitting outcomes and biologically interpretable screened variables.

*LCORL*, a member of the helix‐turn‐helix motif Psq (HTH‐Psq) family, has been genetically linked to phenotypic traits including BH and BW. A 2024 endocrinology study indicated that *LCORL* may function as a regulator of BW and glucose homeostasis in mice. Together with prior research, these findings further suggest that *LCORL* plays a role in the regulation of growth and metabolism across humans and livestock species [24]. Studies have shown that the *LCORL–NCAPG* locus is associated with BW across various physiological stages in cattle [25] and is also related to body size in different horse breeds [26–28]. Yu et al. reported a frameshift mutation in *LCORL* that increases the average daily gain by 71 g in Simmental and Charolais beef cattle from Central Europe. Notably, mutations resulting in the complete loss of the PIP domain of *LCORL* were identified across seven species and were found to exert positive effects on growth performance, albeit to varying degrees and affecting different traits [29]. In addition to the aforementioned effects on growth performance, variants in *LCORL* have been associated with a reduced risk of progression from mild cognitive impairment to Alzheimer’s disease, thereby significantly lowering the susceptibility to Alzheimer’s disease [30]. Given that *LCORL* is associated with height, the aforementioned findings suggest a negative correlation between human height and Alzheimer’s disease, which is consistent with the results reported by Bulik‐Sullivan et al. in their 2015 study [31]. Most studies on *LTBP2* have primarily focused on its roles in cancer and neoplasms [32, 33], and emerging evidence suggests that it may also play a role in delaying organ aging [34]. Studies have also identified *LTBP2* as a critical factor in anterior chamber development, with null mutations in this gene being associated with primary congenital glaucoma [35]. A similar phenomenon has also been documented in Burmese cats [36].

Sun et al. [37] conducted a correlation analysis between BW and body size traits in Tibetan sheep, revealing that CaC, chest circumference, BL, and BH were all significantly correlated with BW. Huang et al. [38] identified significant correlations between slaughter traits and body size traits in lake sheep. A study by Jiang et al. [29] revealed that *LCORL* exerts a positive effect on growth traits in sheep, suggesting its role in contributing to superior growth performance. It is hypothesized that a correlation exists between growth and slaughter performance in Charolais sheep and that *LCORL* may mediate this relationship by influencing both traits. We performed correlation analyses between growth performance and slaughter performance. The results indicated that traits including CC, CD, BL, BH, and WH were significantly or highly significantly correlated with BW. Furthermore, all slaughter‐related traits also showed significant or highly significant correlations with weight. Subsequently, an improved multiple linear regression model was established based on a set of variables identified as strong predictors. An analysis by Jiang et al. [39] on Charolais sheep demonstrated that BW was strongly correlated with several body size measurements. In a correlation study on BW and morphological traits of Kyrgyz sheep at various growth stages, Luo and colleagues [40] reported that chest circumference exhibited the strongest correlation with BW in lambs, whereas CaC was the most correlated trait in rams, and BL showed the highest association in ewes. In line with prior studies, our results confirm the robustness and reference value of the correlation analysis undertaken in this work.

As a globally significant meat sheep breed, Charolais sheep are selected for large body size, a trait with stable heritability. To enhance breed quality, researchers are now prioritizing the development of more stable and superior family lines. This study evaluates the production‐related SNPs in the *LCORL* and *LTBP2* genes from a genetic perspective. The TT genotype was identified as the optimal pleiotropic genotype, and the TTAA haplotype combination was determined to be the most favorable. These findings offer valuable insights for the breeding and genetic improvement of Charolais sheep.

## 5. Conclusion

This study identified a SNP (T136210A) within *LCORL* in Charolais sheep. The results demonstrated that the TT genotype was associated with superior growth and slaughter performance in ewes. In rams, however, the AA genotype correlated with better growth performance, whereas the TT genotype was linked to enhanced slaughter performance. Another (G105452A) was identified in *LTBP2* of Charolais sheep. In terms of growth performance, the AA genotype was dominant in ewes, whereas the GG genotype was predominant in ewe lambs. With respect to slaughter performance, only the GG genotype was observed. In the analysis of *LCORL* and *LTBP2* haplotype combinations, the TTAA haplotype was associated with superior growth performance in ewes, whereas the TTGG combination was linked to improved growth in ewe lambs. For rams and ram lambs, the TAGA haplotype conferred advantages in growth traits. Multivariate linear regression analysis revealed distinct relationships between body measurements and weight across different animal categories. For growth performance, BaH exhibited the strongest direct negative effect on ewe weight (−0.571), while CD showed the greatest direct negative influence on ram weight (−1.858). In lambs, chest circumference had the largest positive direct effect on ewe lamb weight (0.594), whereas hip width was the strongest positive predictor for ram lamb weight (0.518). Regarding slaughter performance, the GR value demonstrated the largest direct positive effect on both ewe weight (1.032) and ram weight (1.395). It is anticipated that the findings of this study will serve as a valuable benchmark for future work.

## Author Contributions

Jiaqi Ge: writing–original draft and software. Ziwen Qi: writing–review and editing. Jianing Xin: writing–review and editing. Yvxin Shi: investigation. Wangshu Li: data curation. Dakun LYu: methodology. Yunlong Guo: data curation. Fengzhi Liu: conceptualization. Decui Xia: formal analysis. Xuehai Du: formal analysis. Guochun Wang: investigation. Shuhuan Yang: investigation. Zeying Wang: supervision and funding acquisition.

## Funding

This work was supported by the 2026 Liaoning Provincial Department of Science and Technology Applied Basic Research Program Project: Research and Demonstration on Key Technologies for Intensive Green Production of Liaoyu Charolais Sheep (Grant No. 2026JH2/101300048); the 2025 Fuxin City Science and Technology Plan Project: Research on Precision Breeding of a High‐Fecundity New Population of Liaoyu Charolais Sheep (No. FX20250211); the 2023 Liaoning Province “Take the Lead” Science and Technology Plan Project (Technical Tackling Category): Breeding of New Varieties of Charbroils Meat Sheep (Grant No. 2023JH1/10400023); and the 2025 Liaoning Province Chief Science and Technology Specialist Program for Meat Sheep (Grant No. 2025JH5/10400199).

## Ethics Statement

This study was approved by the Animal Ethics Committee of Shenyang Agricultural University (Approval No. 2023041610). All procedures were performed in accordance with relevant guidelines and regulations to ensure animal welfare.

## Conflicts of Interest

The authors declare no conflicts of interest.

## Data Availability

The data that support the findings of this study are available from the corresponding author upon reasonable request.
